# Diabetes-specific formula with standard of care improves glycemic control, body composition, and cardiometabolic risk factors in overweight and obese adults with type 2 diabetes: results from a randomized controlled trial

**DOI:** 10.3389/fnut.2024.1400580

**Published:** 2024-07-15

**Authors:** Siew Ling Tey, Winnie S.S. Chee, Chaicharn Deerochanawong, Yatin Berde, Lee-Ling Lim, Apussanee Boonyavarakul, Brittany Wakefield, Geraldine Baggs, Dieu Thi Thu Huynh

**Affiliations:** ^1^Abbott Nutrition Research and Development, Asia-Pacific Centre, Singapore, Singapore; ^2^Department of Nutrition and Dietetics, International Medical University, Kuala Lumpur, Malaysia; ^3^Department of Medicine, Rajavithi Hospital, College of Medicine, Rangsit University, Bangkok, Thailand; ^4^Biostatistics and Statistical Programming, Cognizant Technology Solutions, Mumbai, India; ^5^Department of Medicine, University of Malaya, Kuala Lumpur, Malaysia; ^6^Department of Medicine, Phramongkutklao Hospital, Bangkok, Thailand; ^7^Abbott Nutrition Research and Development, Columbus, OH, United States

**Keywords:** type 2 diabetes, nutrition therapy, meal replacement, diabetes-specific formula, glycemic control, body composition, cardiometabolic risk factors

## Abstract

**Background and aims:**

Medical nutrition therapy is important for diabetes management. This randomized controlled trial investigated the effects of a diabetes-specific formula (DSF) on glycemic control and cardiometabolic risk factors in adults with type 2 diabetes (T2D).

**Methods:**

Participants (*n* = 235) were randomized to either DSF with standard of care (SOC) (DSF group; *n* = 117) or SOC only (control group; *n* = 118). The DSF group consumed one or two DSF servings daily as meal replacement or partial meal replacement. The assessments were done at baseline, on day 45, and on day 90.

**Results:**

There were significant reductions in glycated hemoglobin (−0.44% vs. –0.26%, *p =* 0.015, at day 45; −0.50% vs. −0.21%, *p =* 0.002, at day 90) and fasting blood glucose (−0.14 mmol/L vs. +0.32 mmol/L, *p =* 0.036, at day 90), as well as twofold greater weight loss (−1.30 kg vs. –0.61 kg, *p*  < 0.001, at day 45; −1.74 kg vs. –0.76 kg, *p* < 0.001, at day 90) in the DSF group compared with the control group. The decrease in percent body fat and increase in percent fat-free mass at day 90 in the DSF group were almost twice that of the control group (1.44% vs. 0.79%, *p =* 0.047). In addition, the percent change in visceral adipose tissue at day 90 in the DSF group was several-fold lower than in the control group (−6.52% vs. –0.95%, *p* < 0.001). The DSF group also showed smaller waist and hip circumferences, and lower diastolic blood pressure than the control group (all overall *p* ≤ 0.045).

**Conclusion:**

DSF with SOC yielded significantly greater improvements than only SOC in glycemic control, body composition, and cardiometabolic risk factors in adults with T2D.

## 1 Introduction

Diabetes affected 537 million people (i.e., 10.5% of the population aged 20–79 years) globally in 2021, and by 2045 this figure is projected to reach 783 million (12.2% of the world’s population) (1, 2). Among Western Pacific countries, Malaysia and Thailand ranked in the top 5 countries for diabetes prevalence in 2021 (4.4 million and 6.1 million individuals, respectively) (1). Over 90% of all diabetes cases are attributed to type 2 diabetes (T2D), which is characterized by insulin resistance and progressive deficiency in insulin secretion (1, 3, 4). Overweight and obesity are key modifiable risk factors for T2D (1, 5, 6). Globally, 80–90% of individuals with diabetes are estimated to be overweight or obese (7–10). Among adults in Thailand in 2018, the prevalence of overweight and obesity were 33.2% and 11.6%, respectively; in Malaysia in 2019, 30.4% and 19.7%, respectively (11). Notably, obesity compounds the challenges of glycemic control in T2D. A retrospective study of individuals with T2D showed that people classified as either obese class I or II (body mass index [BMI] 30.0 to <40.0 kg/m^2^) had a greater likelihood of poor glycemic control (glycated hemoglobin [HbA1c] ≥ 7%) than did those classified to have normal weight (BMI 18.5 to <25.0 kg/m^2^) (12).

The American Diabetes Association (ADA) recommends a holistic, multifactorial, person-centered treatment approach that integrates lifestyle modifications (including dietary intervention and physical activity) with pharmacological therapy to improve outcomes in individuals with diabetes (13). For overweight or obese individuals with T2D, the ADA suggests lifestyle changes that help attain and sustain a 5% reduction in body weight by aiming for a daily energy deficit of 500–750 kcal. The guideline also recommends achieving a 3–7% weight reduction for positive effects on glycemic control and managing cardiovascular risk factors in overweight or obese individuals with diabetes (2). Indeed, the Look AHEAD study, which assessed intensive lifestyle intervention through reduced caloric intake and increased physical activity in overweight or obese individuals with T2D, showed that a 5–10% reduction in body weight was associated with reduction in HbA1c, decrease in systolic and diastolic blood pressures, increase in high-density lipoprotein cholesterol (HDL-C), and a reduction in triglycerides (14). Additionally, individuals who experienced remission throughout the follow-up period demonstrated a 33% decrease in the incidence of chronic kidney disease and a 40% decrease in composite cardiovascular disease measure (15). The Diabetes Prevention Program demonstrated a 58% reduction in the risk of developing T2D with an intensive lifestyle intervention (with low-calorie, low-fat diet and physical activity) targeting sustained 7% weight reduction in individuals with prediabetes over 2.8 years, surpassing the effect of metformin (16).

Medical nutrition therapy (MNT) plays a crucial role in diabetes management by helping individuals with prediabetes and T2D adjust their diet to achieve weight loss and optimal glycemic and metabolic control (17, 18). Conventional nutrition therapy with dietary counseling and education has several constraints, including patients’ insufficient understanding of intricate meal structuring, restricted access to nutritious foods, and the challenges of adhering to prescribed diets (19). Meal replacements are prepackaged food products or drinks that are designed to provide a defined amount of energy and replace one or more meals. The ADA Standards of Medical Care in Diabetes recognizes that structured low-calorie meal plans with meal replacements can be safely utilized in the short term (1–2 years) to help individuals with diabetes attain weight loss objectives (20). The nutritional therapy guidance from Diabetes Canada also suggests that incorporating meal replacements (replacing 1 or 2 meals per day), into weight loss programs may be beneficial for individuals with diabetes (21). Similar recommendations are applicable in Asian countries. For example, in Malaysia, the T2D clinical practice guidelines recommend incorporating meal replacement as part of a structured meal plan for weight loss and weight maintenance (6).

Adhering to a diet can be challenging for many individuals, especially those with limited cooking skills and nutritional knowledge. Diabetes-specific formulas (DSFs) are useful strategies to deliver essential macronutrients and micronutrients that are important for individuals with diabetes. DSFs are formulated to contain slowly digestible carbohydrates, healthy fats (monounsaturated fatty acids [MUFAs] or polyunsaturated fatty acids [PUFAs]), protein and fiber, and specific micronutrients (vitamins and minerals) (22, 23). A key advantage for using DSFs is the simplicity of the intervention. DSFs offer several benefits, including ease of preparation, convenience, and built-in portion control, making them a practical and user-friendly dietary option. Furthermore, the low-calorie formulation of DSFs, particularly as meal replacement or partial meal replacement, ensures that individuals with diabetes who are overweight or obese can maintain a calorie deficit sufficient for weight reduction (22, 23).

Medical nutrition therapy is recommended as a part of management strategies for individuals with diabetes in several international guidelines (20, 24, 25). The Transcultural Diabetes Nutrition Algorithm (tDNA) offers a systematic approach to nutritional management for individuals with prediabetes and T2D. It incorporates evidence-based recommendations and is designed to be adaptable to diverse cultures and geographic locations (26). The Asia tDNA and Malaysia tDNA recognize the potential benefits of DSFs in improving glycemic control parameters and suggest DSF inclusion in MNT for the management of prediabetes and T2D (27, 28).

Previous research findings demonstrated the positive impact of meal replacement or partial meal replacement in reducing body weight, improving glycemic control (HbA1c and glucose levels), and managing cardiometabolic risk factors (blood lipids and blood pressure) in individuals with diabetes (22, 23, 29–39). The efficacy of meal replacements is contingent on their unique formulation, which may vary and contribute to different extents of effects on body weight, glycemic control, and cardiometabolic outcomes. Furthermore, variations in the intensity of the interventions may contribute to the differing degrees of impact on the outcomes (22, 23, 40). A current gap in the literature is that the benefits of DSFs in individuals with diabetes were mostly shown by research conducted in Western populations (22, 23, 38, 39). The risk of insulin resistance varies between Asian and Western populations, with the former being more prone to visceral adiposity, in addition to beta-cell dysfunction (41). Some Asian studies of DSF intervention have been reported, but small sample sizes and non-randomized design make it difficult to draw definitive conclusions (29, 32, 37, 42).

This study was done to address these limitations. The objective of this study was to investigate the effects of DSF in addition to standard of care (SOC), as compared with SOC alone, on glycemic control, body composition, and cardiometabolic risk factors in overweight or obese adults with T2D.

## 2 Materials and methods

### 2.1 Study design

This was a 12-week, randomized, parallel study with two treatment arms in a 1:1 ratio: (i) DSF with SOC (‘DSF group’) and (ii) SOC only (‘control group’) were stratified based on the participants’ HbA1c levels (7.0 to <8.0% or 8.0 to <10.0%) and BMI (23.0 to <27.5 kg/m^2^ or 27.5 to <35.0 kg/m^2^) at baseline. The *a priori* specified primary outcome was change in HbA1c level from day 0 to day 90. Other *a priori* specified outcomes included fasting blood glucose, insulin and lipid profiles, anthropometric measurements, body composition, and blood pressure.

### 2.2 Study population

Participants were recruited by the investigators from four main study sites: two in Malaysia and two in Thailand. Recruitment was via hospitals, practices or clinics, referrals, patient database reviews, and advertising.

Participants were eligible for inclusion if they were male or non-pregnant, non-lactating female; aged ≥21 to ≤65 years; with T2D, as evidenced by the use of oral glucose-lowering drug(s); with BMI ≥ 23.0 to <35.0 kg/m^2^ and stable weight during the 2 months prior to the baseline visit; willing to follow the protocol as described; and with a commitment to refrain from taking non-study DSFs throughout the study. Participants were excluded from the study if their screening HbA1c level was <7% or ≥ 10%, if they used exogenous insulin for glucose control, or if they had any of the following conditions: confirmed type 1 diabetes and/or a history of diabetic ketoacidosis, current infection that required medication, inpatient surgery or received systemic corticosteroid treatment in the last 3 months or received antibiotics in the last 3 weeks, active malignancy within the last 5 years, a significant cardiovascular event within 6 months prior to study entry, end-stage organ failure, renal disease, hepatic disease, bariatric surgery, gastrointestinal disease or intestinal surgery, contagious infectious disease, eating disorder, severe dementia or delirium, history of significant neurological or psychiatric disorder, alcoholism, substance use, blood or blood-related diseases, or allergy or intolerance to any ingredient found in the study DSF.

The study was approved by the Malaysia Medical Research and Ethics Committee (NMRR-19-3929-52070) and the Thailand Central Research Ethics Committee (COA-CREC071/2020) and was conducted in accordance with the Declaration of Helsinki. Written informed consent was given by the participants, and the study was prospectively registered at clinicaltrials.gov as NCT04345497, first posted on 14 April 2020.

### 2.3 Study protocol

Eligible participants were randomly allocated to either DSF with SOC (DSF group) or SOC only (control group). Randomization schedules were computer generated using a dynamic minimization algorithm. An electronic data capture system assigned each participant with a unique participant number and randomized them to study treatment according to the generated randomization schedules. The randomization was stratified by participants’ HbA1c level (7.0 to <8.0% or 8.0 to <10.0%) and BMI (23.0 to <27.5 kg/m^2^ or 27.5 to <35.0 kg/m^2^) at baseline. As eligible participants were enrolled, they were sequentially assigned a unique participant number in ascending numerical order within the site and strata combination.

Participants were not blinded to the treatment allocation; however, the study product was labelled with clinical product code and packaged in a plain single-serving sachet, so neither the participants nor the investigators were aware of the details of the study DSF. In addition, the investigators and research staff who performed study outcome assessments were blinded wherever possible to reduce bias. Laboratory personnel who analyzed the blood data were also blinded to the treatment allocation.

All study participants received SOC from a physician-coordinated team, including but not limited to primary care practitioners, physicians or endocrinologists, nurses, dietitians, pharmacists, and diabetes educators (6, 43). The SOC was formulated based on the needs of the participants, as well as the resources available to the researchers. All participants received diabetes education on diet (including food exchanges), exercise, smoking cessation, medication, self-care, and psychosocial adaptation to diabetes. Medical nutrition therapy through healthy food choices was important in managing existing diabetes and delaying complications (2, 20). At each study visit, a dietitian or trained researcher provided dietary counselling according to each participant’s nutritional needs, disease severity, cultural preferences, and willingness to change.

In the control group, participants received only SOC. In the DSF group, in addition to SOC, participants with BMI 23.0 to <27.5 kg/m^2^ were asked to consume one serving of DSF in the morning, while those with BMI 27.5 to <35.0 kg/m^2^ were asked to consume two servings of DSF, one in the morning and one in the evening, as meal replacement or partial meal replacement. Participants in the DSF group were allowed to consume low-glycemic index (GI), low-calorie food in addition to DSF when the DSF was used as partial meal replacement.

The total prescribed daily energy intake was 1,200 to 1,500 kcal for those with BMI 23.0 to <27.5 kg/m^2^, and 1,500 to 1800 kcal for those with BMI 27.5 to <35.0 kg/m^2^. Dietary counselling including healthy food choices, total daily energy intake, and number of DSF servings were adopted from the guidelines for the management of T2D (6, 20, 43) and the tDNA program to optimize diabetes and prediabetes care (26, 28).

The DSF used in this study (Glucerna®; Abbott Nutrition) provided complete and balanced nutrition. It contained a unique low-glycemic carbohydrate blend with sucromalt, a combination of both soluble and insoluble fibers, and a high level of key micronutrients to help manage blood glucose and meet the nutritional needs of people with diabetes. Each serving of the DSF provided 228 kcal, 10.2 g protein, 8.7 g fat predominantly MUFAs and PUFAs, 26.1 g carbohydrate, 800 mg inositol, and 6.51 μg (261 IU) vitamin D_3_ (Supplementary Table S1).

### 2.4 Outcomes

Participants were asked to attend three study visits, where their blood samples, anthropometric measurements, body composition, blood pressure, and physical activity levels were collected at the beginning, middle, and end of the study phase (days 0, 45, and 90). In addition to the study visits, all participants received phone calls from the study team on days 15, 30, and 60, to encourage compliance with the study protocol and to answer queries from the participants.

At baseline, each participant’s socio-demographic and health-related data such as age, gender, ethnicity, education, duration of diabetes, medical history, hospital (re)admission in the last 6 months, and use of medications and nutritional supplements were collected.

At each study visit, fasting blood samples were taken after an overnight fast and sent to a centralized laboratory for the analyses of HbA1c (Cobas c513 analyzer), glucose, insulin, total cholesterol, high-density lipoprotein cholesterol (HDL-C), low-density lipoprotein cholesterol (LDL-C), and triglycerides (Cobas 8,000 modular analyzer). The following formulas were used to calculate homeostasis model assessment of beta-cell function (HOMA-β): (fasting insulin [μU/mL] multiplied by 20) divided by (fasting blood glucose [mmol/L] – 3.5); and homeostasis model assessment of insulin resistance (HOMA-IR): (fasting blood glucose [mmol/L] multiplied by fasting insulin [μU/mL]) divided by 22.5 (44).

Anthropometric measurements (i.e., weight, waist circumference, and hip circumference) were taken at each visit. Height was measured to the nearest millimeter at baseline with a stadiometer. Weight was measured with a weighing scale; the participants wore light clothing and no footwear. BMI [weight (kg) divided by height (m^2^)] was a measurement of weight relative to height that applied to both adult men and women. A medical body composition analyzer (Seca mBCA 525) was used to estimate fat mass, fat-free mass, visceral adipose tissue, total body water, and phase angle. Waist circumference was measured in duplicate using an anthropometric tape at the smallest circumference between the iliac crest and the rib cage; hip circumference, at the maximum protuberance of the buttocks. Blood pressure was measured in triplicate using a blood pressure monitor (Omron HEM-907), and then the readings were averaged.

At every study visit, each participant’s level of physical activity (i.e., vigorous activities, moderate activities, walking, and sitting) over the last 7 days was measured using the short form of the International Physical Activity Questionnaire (45), to determine whether there was any change in the participants’ physical activity levels which might lead to changes in body weight and glycemic control.

Compliance within the DSF group was assessed by reviewing product consumption diaries and by returns of unused product sachets. Percent of compliance was calculated with the following formula: [(number of sachets consumed) divided by (number of sachets instructed to consume)] multiplied by 100. Hedonic rating of the study product was measured using a 9-point hedonic scale at baseline, mid-study, and end of study. The 9-point hedonic scale consisted of “dislike extremely”, “dislike very much”, “dislike moderately”, “dislike slightly”, “neither like nor dislike”, “like slightly”, “like moderately”, “like very much”, and “like extremely”.

Adverse events (AEs) were reported by participants and confirmed by a physician. All reported non-serious and serious AE diagnoses were standardized using the Medical Dictionary of Regulatory Activities version 26. The incidence of all AEs was monitored throughout the study. Additionally, participants were contacted 3 to 7 days after discontinuation of product consumption or study exit, to assess whether any new AEs occurred.

### 2.5 Statistical analysis

The power analysis was based on HbA1c data from Chee et al. (31). To detect the difference seen in the Chee study (−0.3 for the conventional counseling group, compared with 0.1 for the control group, at 3 months) with 80% power using a two-sided 0.05 level t-test assuming a common SD of 0.96%, the required sample size was 92 per group. Assuming a 26% attrition rate, approximately 250 participants (125 per group) were enrolled. All power analyses were conducted using nQuery Advisor, V7 (Statsols, Boston, MA, United States).

For baseline demographic and clinical characteristics of study participants, categorical variables were analyzed using tests of association (Chi-square or Fisher’s exact test), while continuous variables were analyzed using parametric analysis (ANOVA) except when variable distribution was declared non-normal, in which case, non-parametric analysis was used.

*A priori* analysis used analysis of covariance (ANCOVA) with factors for treatment group, site, randomization stratum BMI, and baseline HbA1c value to determine the change in HbA1c from day 0 to day 90 (primary outcome) between the groups. Changes in fasting blood glucose, body weight, fat mass, fat-free mass, visceral adipose tissue, and HOMA-β from day 0 to subsequent timepoints were analyzed using ANCOVA with factors for treatment group, site, randomization strata HbA1c level and BMI, and baseline value for the specific outcome variable. Post-hoc analysis was conducted using the same method to compare the effects of incorporating DSF at two different doses (one versus two servings per day; intervention group) with a diet without DSF (control group) on HbA1c, body weight, and visceral adipose tissue. Stepdown Bonferroni (Holm) *p* value adjustments were made to account for multiple comparisons. In addition, subgroup analysis was done based on participant’s baseline BMI status (23.0 to <27.5 kg/m^2^ or 27.5 to <35.0 kg/m^2^). The same method was used to determine the differences in blood lipid profiles between the groups.

All the outcomes at day 45 and day 90 were analyzed using repeated measures ANCOVA with factors for treatment group, site, randomization strata HbA1c level and BMI, visit, treatment group by visit interaction, and baseline value for the specific outcome variable. Stepdown Bonferroni (Holm) *p* value adjustments were made to account for multiple comparisons. The “overall” results (treatment main effect) and “by visit” results (treatment group by visit interaction effect) were obtained from the same repeated measures ANCOVA.

All analyses were done using the modified intent-to-treat (MITT) dataset, which was defined as all available data from all participants, as well as DSF group participants who received at least one study feeding. A total of 229 out of 235 randomized participants (97.4%) were included in the MITT analysis, and 219 participants (93.2%) were included in the per-protocol analysis. Results from the per-protocol analysis (*n* = 219) confirmed the MITT results. Thus, only MITT results are presented in this manuscript. Missing values were not imputed due to the low attrition rate. SAS version 9.4 (SAS Institute, Cary, NC, United States) was used for all statistical analyses, and *p* < 0.05 was considered statistically significant.

## 3 Results

### 3.1 Study population

**Figure 1:**
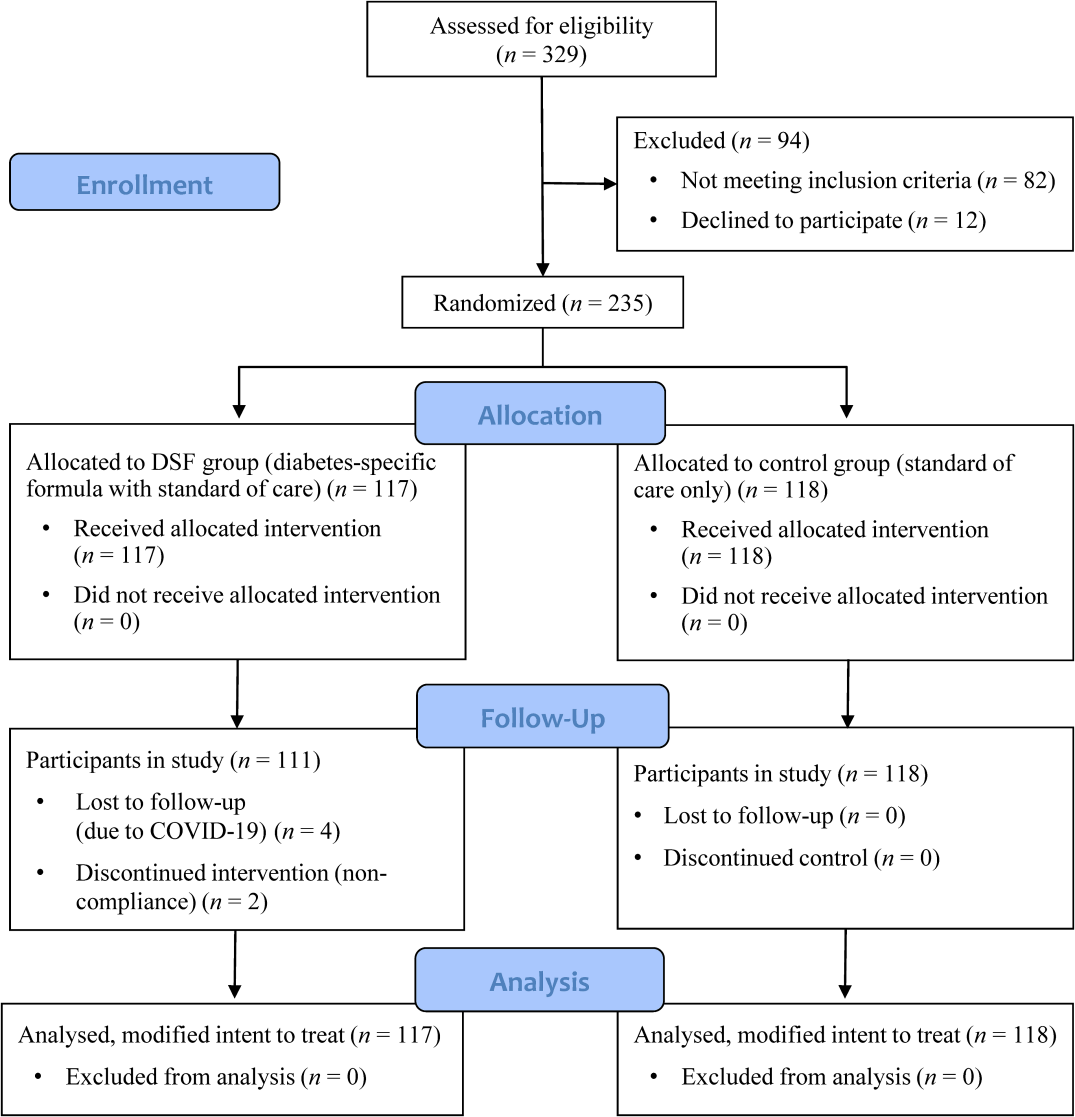
Participant flow chart.

Figure 1 shows the participant flow chart. Participants were enrolled into the study between August 2020 and September 2022, with a 3-month intervention period from enrollment. A total of 329 adult men and women with T2D were screened for eligibility: 82 did not meet the inclusion criteria, 12 declined to participate, and 235 were randomized to the DSF group (DSF with SOC) (*n* = 117) or the control group (SOC only) (*n* = 118). Four participants in the DSF group withdrew from the study due to reasons related to COVID-19, and a further two participants discontinued the intervention. All randomized participants were included in the MITT analysis (Figure 1).

Table 1 shows the baseline demographic and clinical characteristics of the study participants. At baseline, approximately 41% were males and 59% were females. Mean (SE) age of the participants was 54.0 (0.5) years, BMI was 28.37 (0.21) kg/m^2^, and HbA1c level was 7.94 (0.05)% (Table 1). Baseline body composition, blood pressure, and biochemical outcomes are presented in Supplementary Table S2.

**Table 1:** Baseline demographic and clinical characteristics of study participants.

	All participants (*n* = 235)	DSF (*n* = 117)	Control (*n* = 118)	*p*-value
*Gender*				0.203
Male	96 (40.9)	43 (36.8)	53 (44.9)	
Female	139 (59.1)	74 (63.2)	65 (55.1)	
Age (years)	54.0 ± 0.5	54.2 ± 0.8	53.8 ± 0.7	0.623
Ethnic group, *n* (%)				0.842
Chinese	42 (17.9)	18 (15.4)	24 (20.3)	
Malay	36 (15.3)	18 (15.4)	18 (15.3)	
Indian	66 (28.1)	36 (30.8)	30 (25.4)	
Thai	87 (37.0)	43 (36.8)	44 (37.3)	
Other	4 (1.7)	2 (1.7)	2 (1.7)	
Highest level of education, *n* (%)				0.727
No formal education	8 (3.4)	6 (5.1)	2 (1.7)	
Primary school or equivalent	36 (15.3)	17 (14.5)	19 (16.1)	
Secondary or middle school or equivalent	107 (45.5)	53 (45.3)	54 (45.8)	
Diploma or high school or equivalent	40 (17.0)	20 (17.1)	20 (16.9)	
University & above	44 (18.7)	21 (17.9)	23 (19.5)	
Height (cm)	160.78 ± 0.58	159.66 ± 0.72	161.89 ± 0.91	0.046
Body weight (kg)	73.54 ± 0.76	72.59 ± 0.94	74.47 ± 1.18	0.138
BMI (kg/m^2^)	28.37 ± 0.21	28.45 ± 0.30	28.29 ± 0.30	0.705
BMI category, *n* (%)				0.956
23.0 to <27.5	98 (41.7)	49 (41.9)	49 (41.5)	
27.5 to <35.0	137 (58.3)	68 (58.1)	69 (58.5)	
Hip circumference (cm)	101.84 ± 0.50	101.97 ± 0.74	101.72 ± 0.66	0.751
Waist circumference (cm)	96.17 ± 0.64	95.72 ± 0.89	96.62 ± 0.93	0.376
HbA1c category, *n* (%)				0.837
>7.0 to 8.0%	133 (56.6)	67 (57.3)	66 (55.9)	
>8.0 to <10.0%	102 (43.4)	50 (42.7)	52 (44.1)	
HbA1c (%)	7.94 ± 0.05	7.93 ± 0.06	7.95 ± 0.07	0.821
Fasting blood glucose (mmol/L)	7.59 ± 0.12	7.54 ± 0.18	7.64 ± 0.17	0.665
Diabetes duration (years)	9.2 ± 0.4	10.0 ± 0.6	8.4 ± 0.5	0.041
History of gestational diabetes, *n* (%)				0.671
Yes	30 (21.6)	17 (23.0)	13 (20.0)	
No	109 (78.4)	57 (77.0)	52 (80.0)	
Number of hospital admissions in the last 6 months				1.000
None	229 (97.4)	114 (97.4)	115 (97.5)	
1	5 (2.1)	3 (2.6)	2 (1.7)	
2	1 (0.4)	0 (0.0)	1 (0.8)	
>2	0 (0.0)	0 (0.0)	0 (0.0)	
*Glucose-lowering drugs* ^*^ *, n (%)*				
Metformin	231 (98.3)	116 (99.1)	115 (97.5)	0.622
Sulfonylureas	144 (61.3)	72 (61.5)	72 (61.0)	1.000
SGLT-2 inhibitors	43 (18.3)	19 (16.2)	24 (20.3)	0.500
DPP-4 inhibitors	44 (18.7)	23 (19.7)	21 (17.8)	0.741
Thiazolidinediones	34 (14.5)	17 (14.5)	17 (14.4)	1.000
GLP-1 analogues	9 (3.8)	4 (3.4)	5 (4.2)	1.000
Alpha glucosidase inhibitor	4 (1.7)	2 (1.7)	2 (1.7)	1.000
IPAQ total physical activity MET min per week	3374.8 ± 284.7	3545.5 ± 370. 6	3197.8 ± 435.5	0.571

For continuous variables, mean values ± SEM are reported; for categorical variables, *n* (%) is reported. For some variables, sample sizes are smaller than the overall stated sample sizes. ^*^Participants may be taking multiple drugs. BMI, body mass index; DPP-4, dipeptidyl peptidase 4; DSF, diabetes-specific formula; GLP-1, glucagon-like peptide-1; HbA1c, glycated hemoglobin; IPAQ, International Physical Activity Questionnaire; MET, metabolic equivalent task; SEM, standard error of mean; SGLT-2, sodium-glucose cotransporter 2.

### 3.2 All outcome measurements at day 45 and day 90

Table 2 shows the anthropometry, body composition, blood pressure, and biochemical outcomes at day 45 and day 90 for both the DSF group and the control group. Body weight, BMI, hip circumference, and waist circumference were significantly lower in the DSF group than in the control group at day 45 (all *p* ≤ 0.028) and day 90 (all *p* ≤ 0.003). Fat mass and visceral adipose tissue were also significantly lower in the DSF group (both overall *p* ≤ 0.004), as were systolic blood pressure at day 90 (*p* = 0.043) and diastolic blood pressure (overall *p* = 0.045) (Table 2).

**Table 2:** Anthropometry, body composition, blood pressure, and biochemical outcomes at day 45 and day 90.

Variables	Overall Days 45 and 90			By visit Day 45			By visit Day 90		
	DSF (*n* = 112)	Control (*n* = 118)	*p*-value	DSF (*n* = 112)	Control (*n* = 118)	*p*-value	DSF (*n* = 111)	Control (*n* = 118)	*p*-value
*Anthropometry*									
Body weight (kg)	72.10 ± 0.15	72.93 ± 0.15	**<0.001**	72.28 ± 0.14	72.97 ± 0.14	**<0.001**	71.92 ± 0.18	72.88 ± 0.18	**<0.001**
BMI (kg/m^2^)	27.77 ± 0.06	28.08 ± 0.06	**<0.001**	27.84 ± 0.05	28.09 ± 0.05	**<0.001**	27.70 ± 0.07	28.06 ± 0.07	**<0.001**
Hip circumference (cm)	100.24 ± 0.21	101.03 ± 0.20	**0.003**	100.57 ± 0.19	101.11 ± 0.19	**0.028**	99.91 ± 0.24	100.95 ± 0.24	**0.003**
Waist circumference (cm)	94.00 ± 0.23	95.04 ± 0.22	**<0.001**	94.28 ± 0.22	95.11 ± 0.22	**0.003**	93.72 ± 0.26	94.97 ± 0.25	**<0.001**
*Body composition*									
Fat mass (kg)	25.91 ± 0.17	26.53 ± 0.17	**0.004**	26.19 ± 0.18	26.57 ± 0.17	0.092	25.64 ± 0.19	26.49 ± 0.19	**0.002**
Fat mass (%)	36.27 ± 0.21	36.70 ± 0.21	0.111	36.56 ± 0.23	36.71 ± 0.23	0.608	35.98 ± 0.25	36.70 ± 0.25	0.058
Fat-free mass (kg)	46.05 ± 0.17	46.09 ± 0.16	0.832	45.95 ± 0.19	46.10 ± 0.18	1.000	46.14 ± 0.20	46.08 ± 0.19	1.000
Fat-free mass (%)	63.73 ± 0.21	63.30 ± 0.21	0.111	63.44 ± 0.23	63.29 ± 0.23	0.606	64.02 ± 0.25	63.30 ± 0.25	0.058
Visceral adipose tissue (L)	2.97 ± 0.03	3.08 ± 0.03	**0.002**	3.00 ± 0.03	3.08 ± 0.03	**0.037**	2.93 ± 0.03	3.08 ± 0.03	**<0.001**
Total body water (%)	47.11 ± 0.15	46.83 ± 0.15	0.156	46.89 ± 0.17	46.84 ± 0.16	0.821	47.33 ± 0.18	46.82 ± 0.18	0.061
Phase angle (°)	5.78 ± 0.03	5.74 ± 0.03	0.372	5.79 ± 0.03	5.73 ± 0.03	0.340	5.77 ± 0.04	5.75 ± 0.04	0.739
*Blood pressure*									
Systolic blood pressure (mmHg)	127.5 ± 0.9	129.6 ± 0.9	0.082	127.8 ± 1.0	128.6 ± 1.0	0.518	127.3 ± 1.1	130.5 ± 1.0	**0.043**
Diastolic blood pressure (mmHg)	76.9 ± 0.6	78.4 ± 0.6	**0.045**	76.5 ± 0.6	78.2 ± 0.6	0.101	77.3 ± 0.7	78.6 ± 0.7	0.159
*Biochemical outcomes*									
HbA1c (%)	7.45 ± 0.06	7.69 ± 0.06	**0.003**	7.50 ± 0.06	7.68 ± 0.06	**0.015**	7.41 ± 0.07	7.70 ± 0.07	**0.004**
Blood glucose (mmol/L)	7.63 ± 0.14	7.92 ± 0.14	0.117	7.80 ± 0.17	7.91 ± 0.16	0.609	7.45 ± 0.17	7.92 ± 0.16	0.066
Insulin (μIU/mL)	15.62 ± 0.72	14.80 ± 0.71	0.373	15.68 ± 0.85	15.22 ± 0.84	0.681	15.57 ± 0.78	14.38 ± 0.77	0.484
HOMA-β	97.62 ± 8.42	85.08 ± 8.27	0.248	93.74 ± 10.06	91.59 ± 9.84	0.871	101.50 ± 8.62	78.56 ± 8.46	0.082
HOMA-IR	5.20 ± 0.29	5.28 ± 0.28	0.829	5.38 ± 0.36	5.34 ± 0.35	1.000	5.01 ± 0.30	5.21 ± 0.30	1.000
Total cholesterol (mmol/L)	4.18 ± 0.06	4.26 ± 0.05	0.245	4.16 ± 0.07	4.25 ± 0.07	0.681	4.19 ± 0.06	4.27 ± 0.06	0.681
Triglycerides (mmol/L)	1.78 ± 0.07	1.89 ± 0.07	0.250	1.73 ± 0.08	1.87 ± 0.08	0.379	1.84 ± 0.08	1.91 ± 0.08	0.484
HDL cholesterol (mmol/L)	1.19 ± 0.01	1.20 ± 0.01	0.470	1.17 ± 0.02	1.20 ± 0.02	0.307	1.21 ± 0.02	1.21 ± 0.02	0.952
LDL cholesterol (mmol/L)	2.19 ± 0.05	2.23 ± 0.05	0.516	2.21 ± 0.06	2.24 ± 0.06	0.896	2.17 ± 0.05	2.22 ± 0.05	0.896
Total cholesterol:HDL cholesterol ratio	3.62 ± 0.07	3.76 ± 0.07	0.124	3.65 ± 0.08	3.76 ± 0.08	0.313	3.59 ± 0.08	3.76 ± 0.07	0.176
IPAQ total physical activity (MET-min per week)	3601.1 ± 342.9	3953.4 ± 346.8	0.421	3657.8 ± 367.4	4449.3 ± 367.4	0.191	3544.4 ± 416.7	3457.4 ± 424.9	0.875

Repeated measures ANCOVA, adjusting for treatment group, site, randomization strata HbA1c level and BMI, visit, treatment group by visit interaction, and baseline value for the specific outcome variable. Stepdown Bonferroni (Holm) *p* value adjustments were made to account for multiple comparisons. The “Overall” column shows the results from the treatment main effect and the “By visit” columns show the results of the treatment group by visit interaction effect. Both results were obtained from the same repeated measures ANCOVA. Values are LSM ± SE. Statistically significant findings (*p* < 0.05) are in bold. For some variables, sample sizes are smaller than the overall stated sample sizes. ANCOVA, analysis of covariance; BMI, body mass index; HbA1c, glycated hemoglobin; HDL, high-density lipoprotein; HOMA-β, homeostasis model assessment of β-cell function; HOMA-IR, homeostasis model assessment of insulin resistance; IPAQ, International Physical Activity Questionnaire; LDL, low-density lipoprotein; LSM, least square mean; MET, metabolic equivalent task; SE, standard error.

### 3.3 Changes in biochemical outcomes, body weight and composition

**Figure 2:**
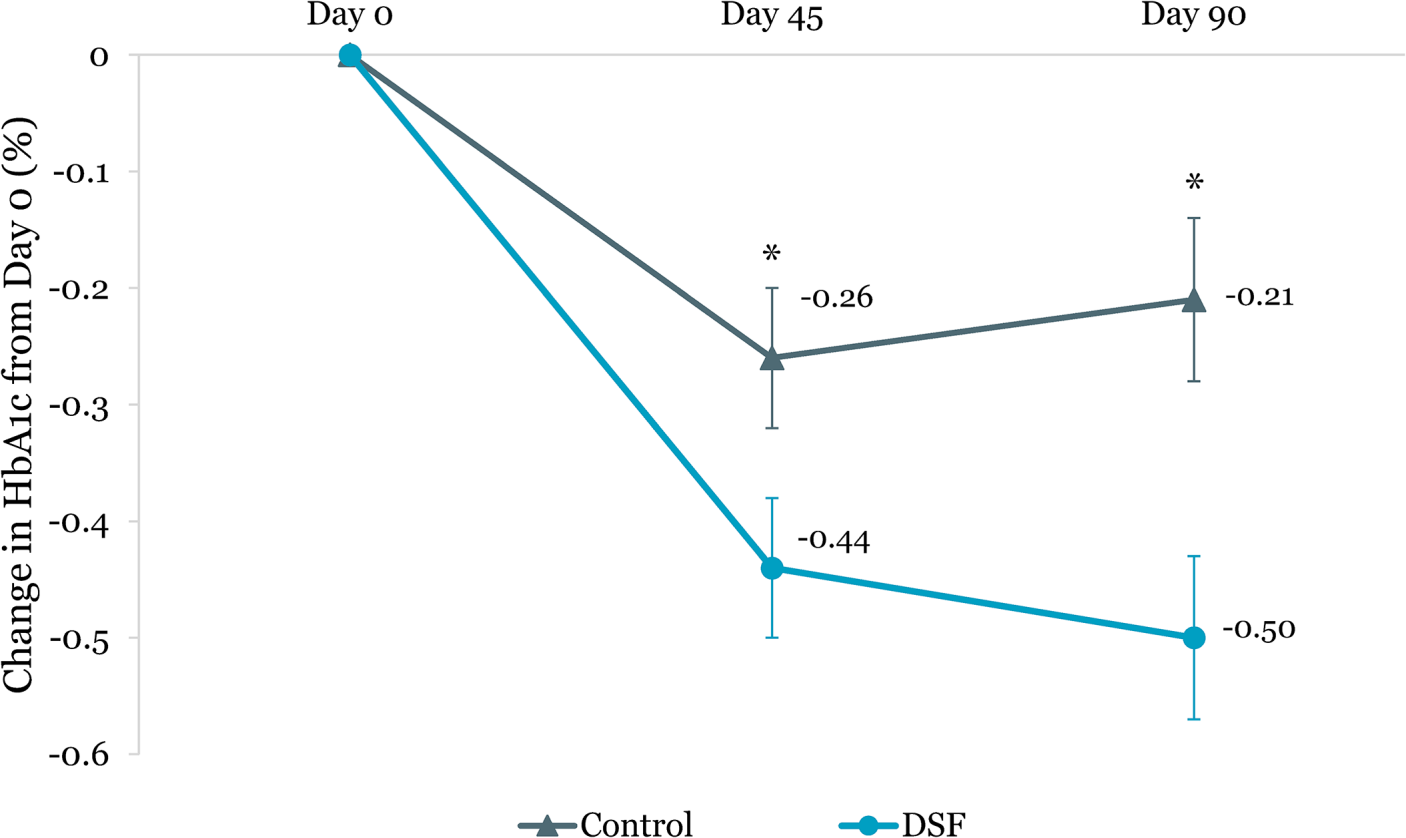
Change in HbA1c (%) from day 0. Values are LSM ± SE. *Significantly different between the groups (*p <* 0.05). DSF, diabetes-specific formula; HbA1c, glycated hemoglobin.

**Figure 3:**
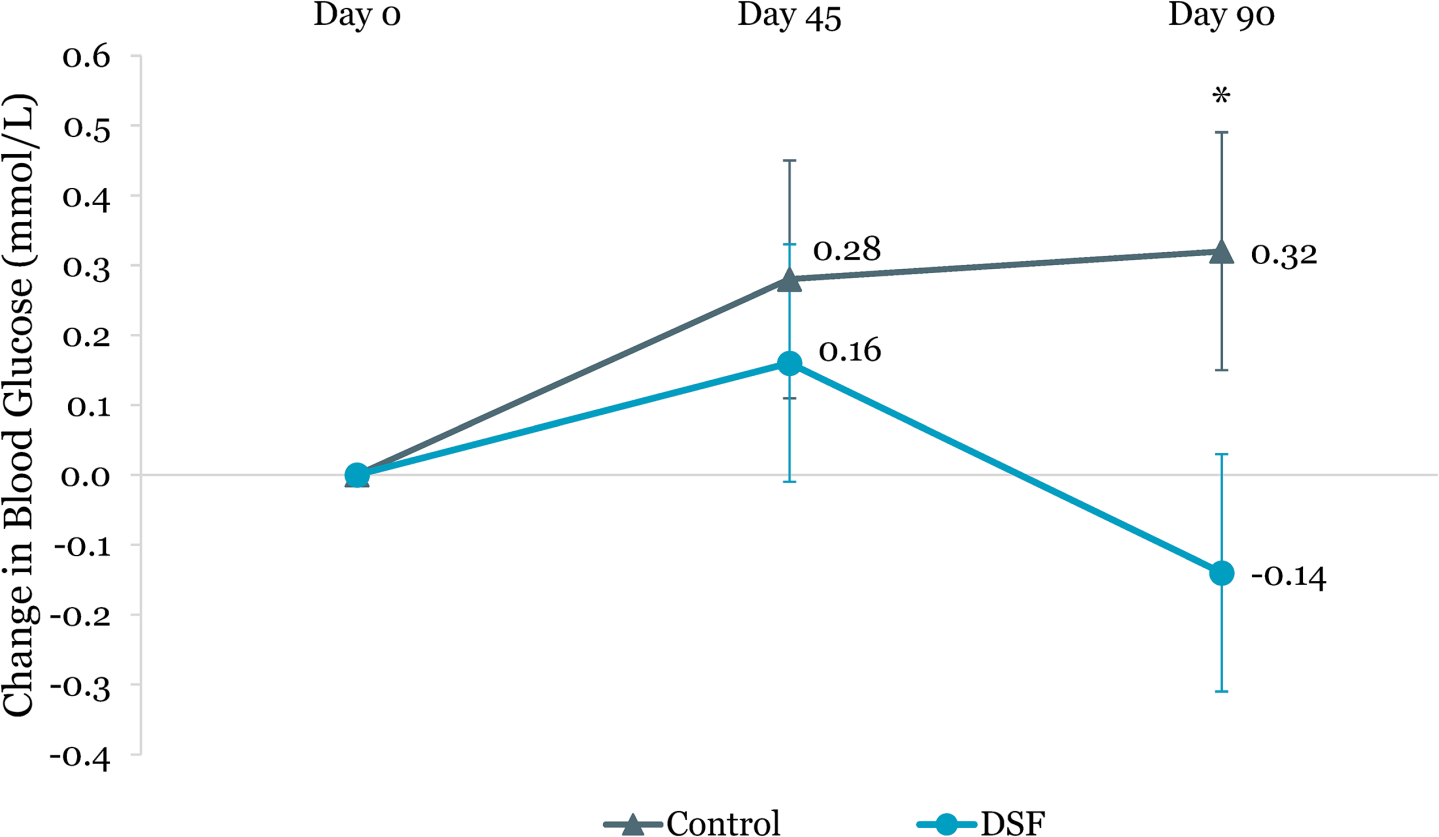
Change in fasting blood glucose (mmol/L) from day 0. Values are LSM ± SE. *Significantly different between the groups (*p <* 0.05). DSF, diabetes-specific formula.

Table 3 shows the changes in HbA1c, blood glucose, body weight and composition from day 0 to day 45 and day 90. The DSF group had a greater HbA1c reduction than the control group at day 45 (−0.44% vs. −0.26%, *p =* 0.015) and day 90 (−0.50% vs. −0.21%, *p =* 0.002) (Figure 2). Fasting blood glucose decreased by 0.14 mmol/L from day 0 to day 90 in the DSF group, whereas it increased in the control group by 0.32 mmol/L (*p =* 0.036) (Figure 3).

**Table 3:** Change in HbA1c, blood glucose, body weight and composition from day 0 to day 45 and day 90.

	Change from day 0 to day 45			Change from day 0 to day 90		
	DSF (*n* = 112)	Control (*n* = 118)	*p*-value	DSF (*n* = 111)	Control (*n* = 118)	*p*-value
HbA1c (%)	−0.44 ± 0.06	−0.26 ± 0.06	**0.015**	−0.50 ± 0.07	−0.21 ± 0.07	**0.002**
Blood glucose (mmol/L)	0.16 ± 0.17	0.28 ± 0.17	0.593	−0.14 ± 0.17	0.32 ± 0.17	**0.036**
Body weight (kg)	−1.30 ± 0.14	−0.61 ± 0.14	**<0.001**	−1.74 ± 0.19	−0.76 ± 0.18	**<0.001**
Body weight (%)	−1.72 ± 0.19	−0.82 ± 0.18	**<0.001**	−2.27 ± 0.25	−1.05 ± 0.24	**<0.001**
Fat mass (kg)	−1.13 ± 0.18	−0.72 ± 0.18	0.070	−1.77 ± 0.20	−0.96 ± 0.19	**0.001**
Fat mass (%)	−0.82 ± 0.23	−0.63 ± 0.23	0.509	−1.44 ± 0.26	−0.79 ± 0.26	**0.047**
Fat-free mass (kg)	−0.16 ± 0.19	−0.02 ± 0.18	0.547	0.0001 ± 0.20	−0.05 ± 0.20	0.845
Fat-free mass (%)	0.82 ± 0.23	0.63 ± 0.23	0.507	1.44 ± 0.26	0.79 ± 0.26	**0.047**
Visceral adipose tissue (L)	−0.16 ± 0.03	−0.08 ± 0.03	**0.039**	−0.23 ± 0.03	−0.07 ± 0.03	**<0.001**
Visceral adipose tissue (%)	−4.46 ± 1.27	−1.65 ± 1.22	0.080	−6.52 ± 1.28	−0.95 ± 1.22	**<0.001**

Analysis of covariance (ANCOVA), adjusting for treatment group, site, randomization strata HbA1c level and BMI, and baseline value for the specific outcome variable. Values are LSM ± SE. Statistically significant findings (*p* < 0.05) are in bold. For some variables, sample sizes are smaller than the overall stated sample sizes. HbA1c, glycated hemoglobin; LSM, least square mean; SE, standard error.

**Figure 4:**
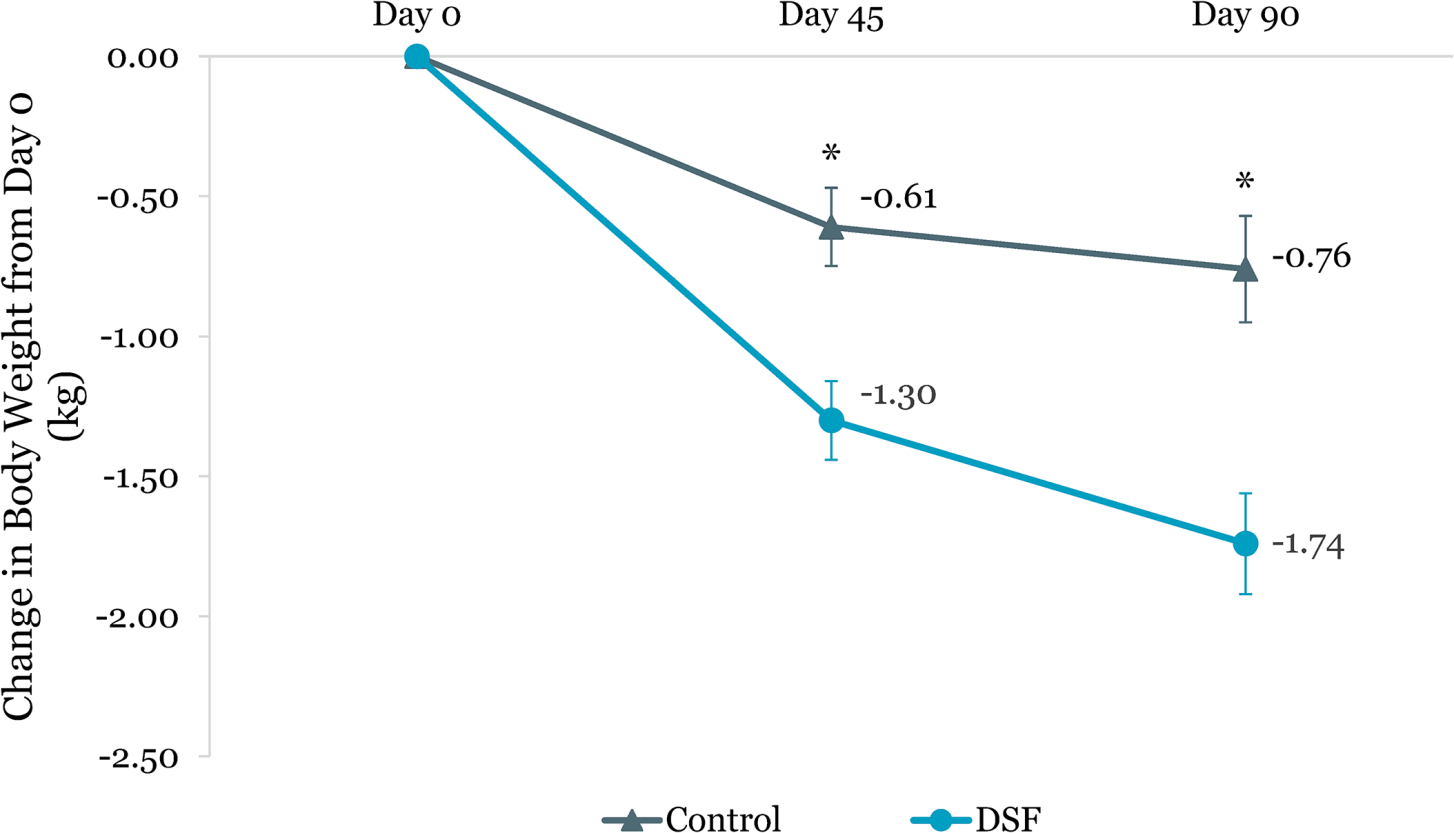
Change in body weight (kg) from day 0. Values are LSM ± SE. *Significantly different between the groups (*p <* 0.05). DSF, diabetes-specific formula.

**Figure 5:**
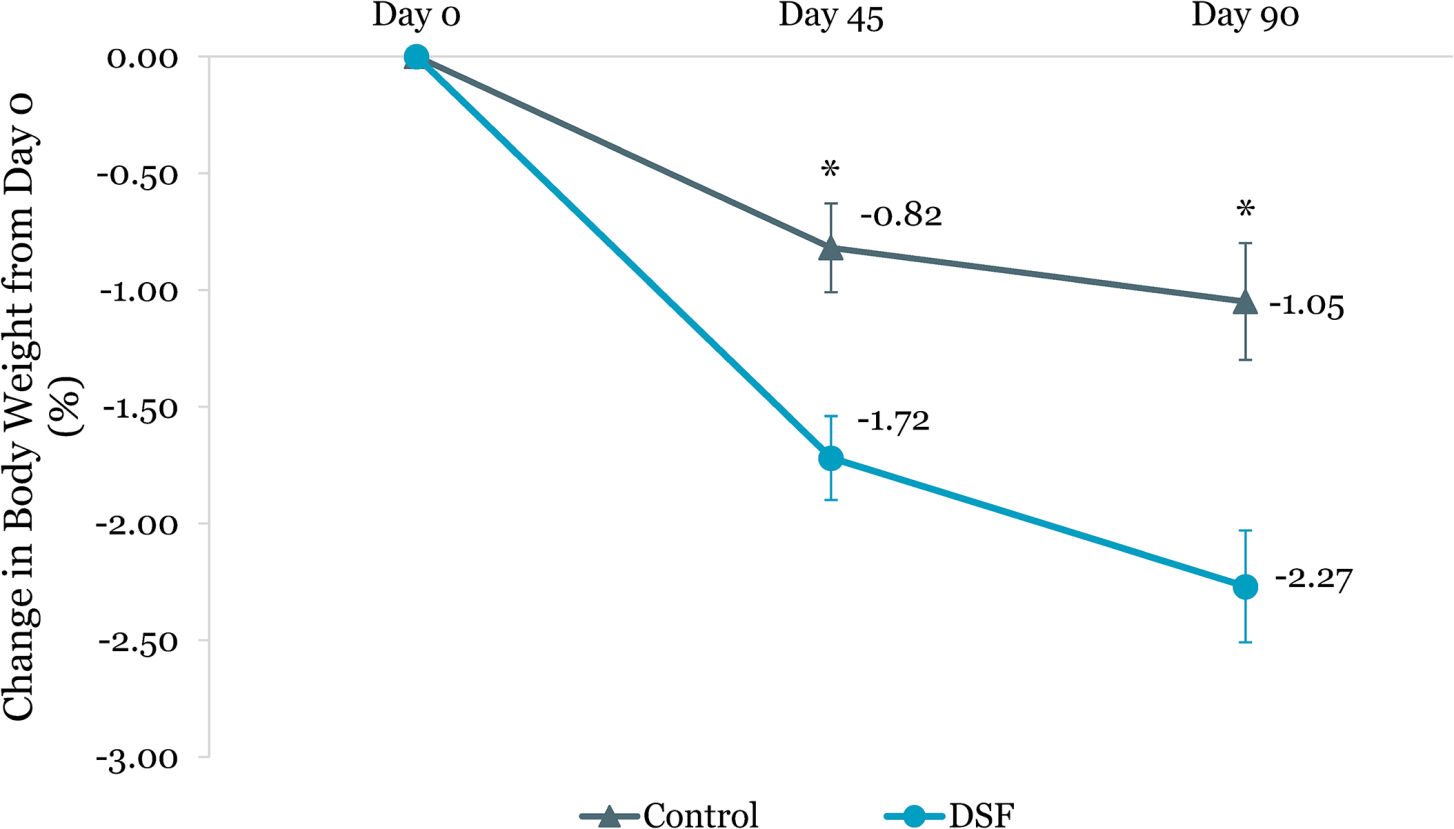
Change in body weight (%) from day 0. Values are LSM ± SE. *Significantly different between the groups (*p <* 0.05). DSF, diabetes-specific formula.

**Figure 6:**
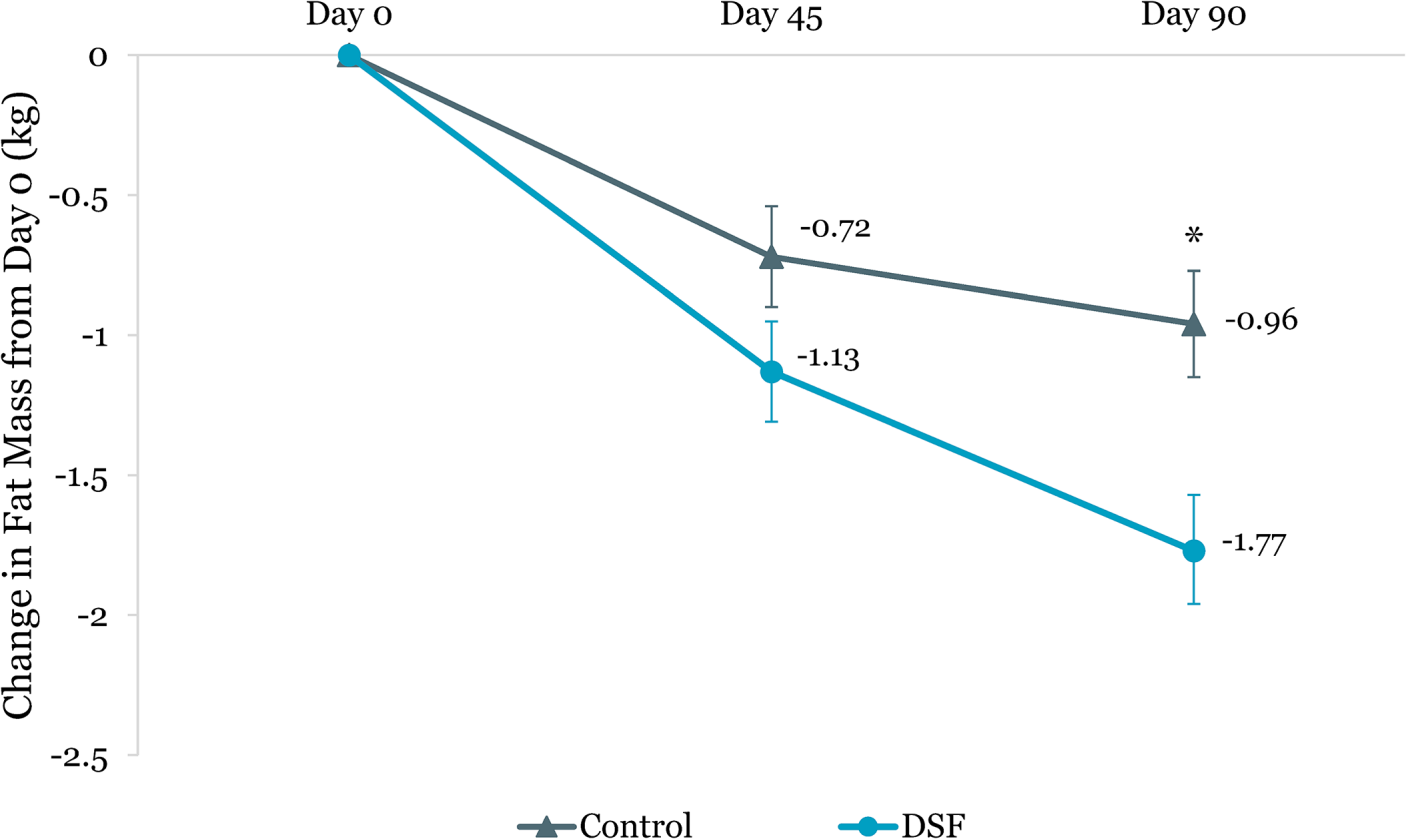
Change in fat mass (kg) from day 0. Values are LSM ± SE. *Significantly different between the groups (*p <* 0.05). DSF, diabetes-specific formula.

**Figure 7:**
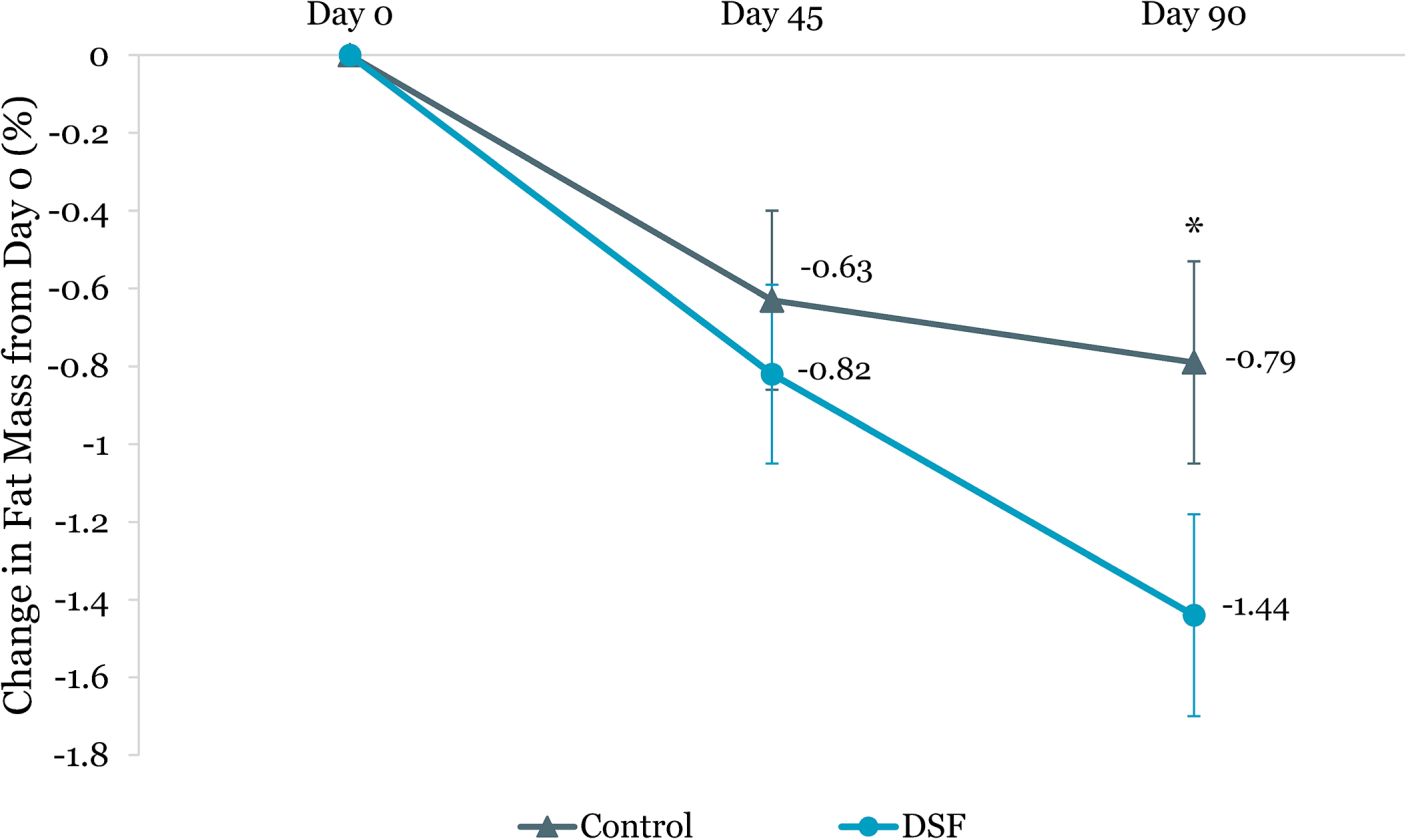
Change in fat mass (%) from day 0. Values are LSM ± SE. *Significantly different between the groups (*p <* 0.05). DSF, diabetes-specific formula.

**Figure 8:**
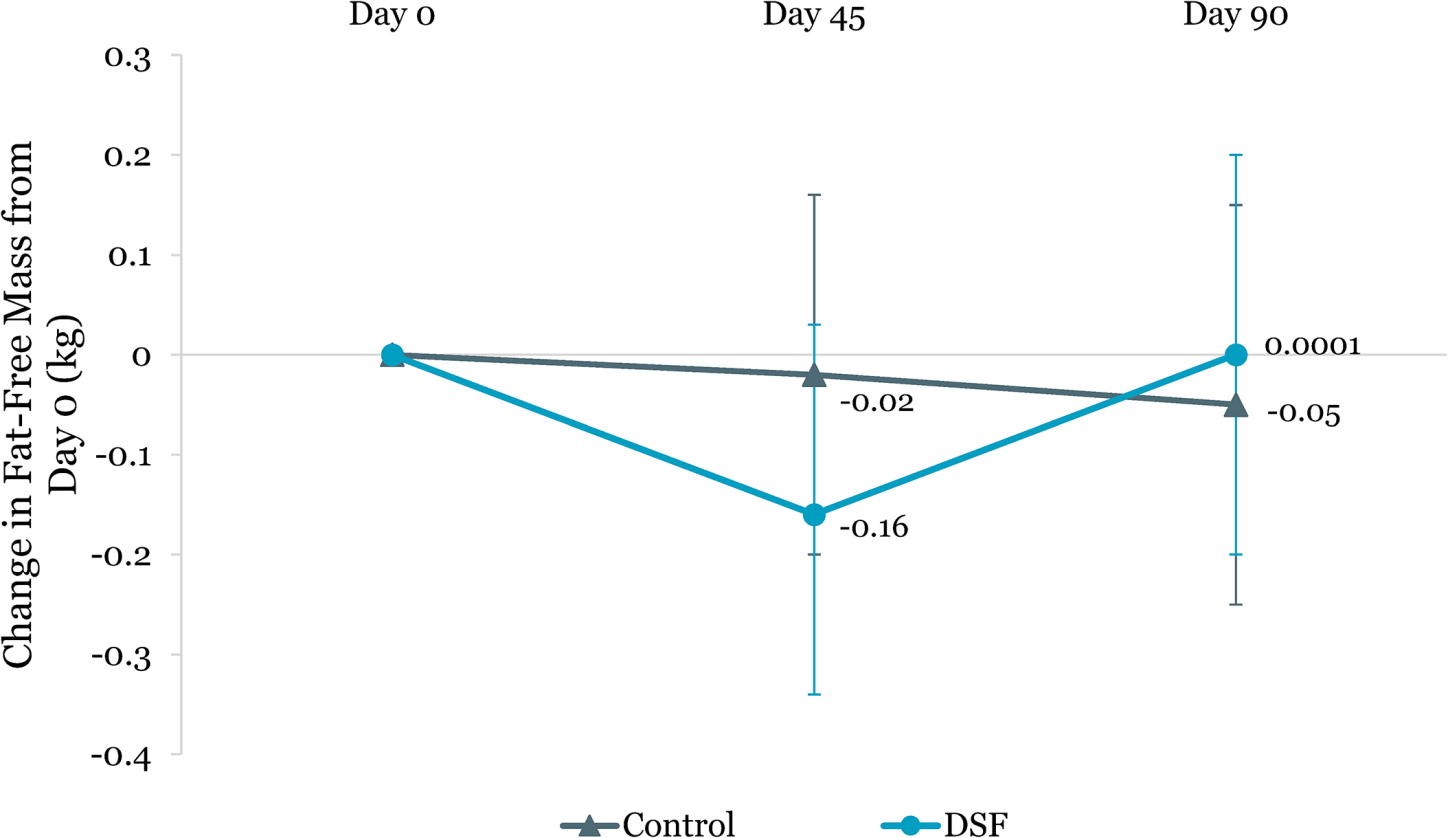
Change in fat-free mass (kg) from day 0. Values are LSM ± SE. *Significantly different between the groups (*p <* 0.05). DSF, diabetes-specific formula.

**Figure 9:**
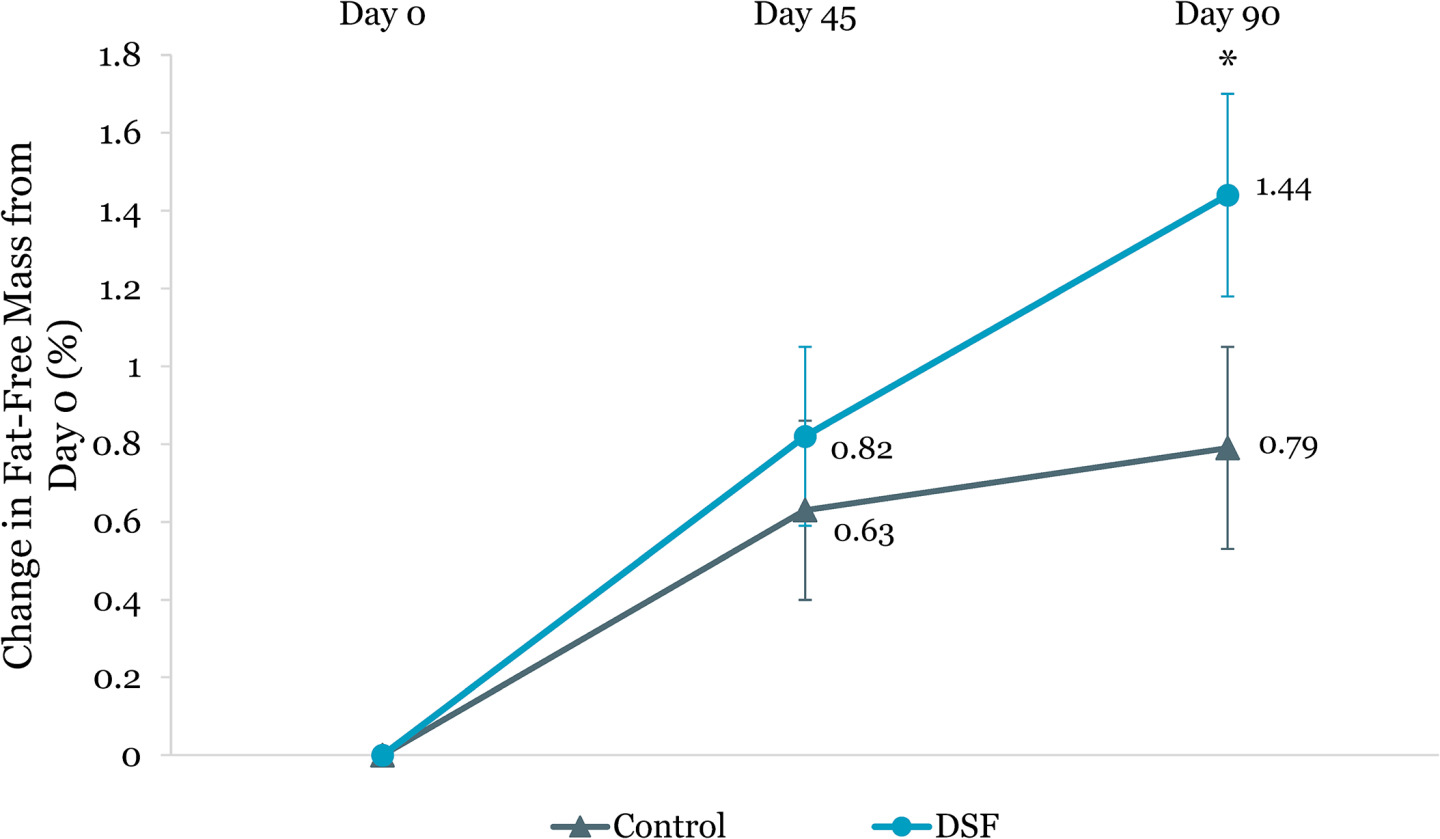
Change in fat-free mass (%) from day 0. Values are LSM ± SE. *Significantly different between the groups (*p <* 0.05). DSF, diabetes-specific formula.

Body weight reduction in the DSF group was twice that of the control group at day 45 (−1.30 kg vs. −0.61 kg, *p <* 0.001) and day 90 (−1.74 kg vs. −0.76 kg, *p <* 0.001) (Figure 4). Similar findings were observed for percent body weight change at day 45 (−1.72% vs. −0.82%, *p <* 0.001) and day 90 (−2.27% vs. −1.05%, *p <* 0.001) (Figure 5). The reductions in fat mass (−1.77 kg vs. −0.96 kg, *p* = 0.001) (Figure 6) and percent body fat (−1.44% vs. −0.79%, *p* = 0.047) (Figure 7) from day 0 to day 90 in the DSF group were significantly greater than those in the control group. Figures 8, 9 show the changes in fat-free mass from baseline to subsequent timepoints, expressed in kilogram and percentage. The increase in fat-free mass (%) from day 0 to day 90 in the DSF group was almost twice that of the control group (1.44% vs. 0.79%, *p* = 0.047) (Figure 9).

**Figure 10:**
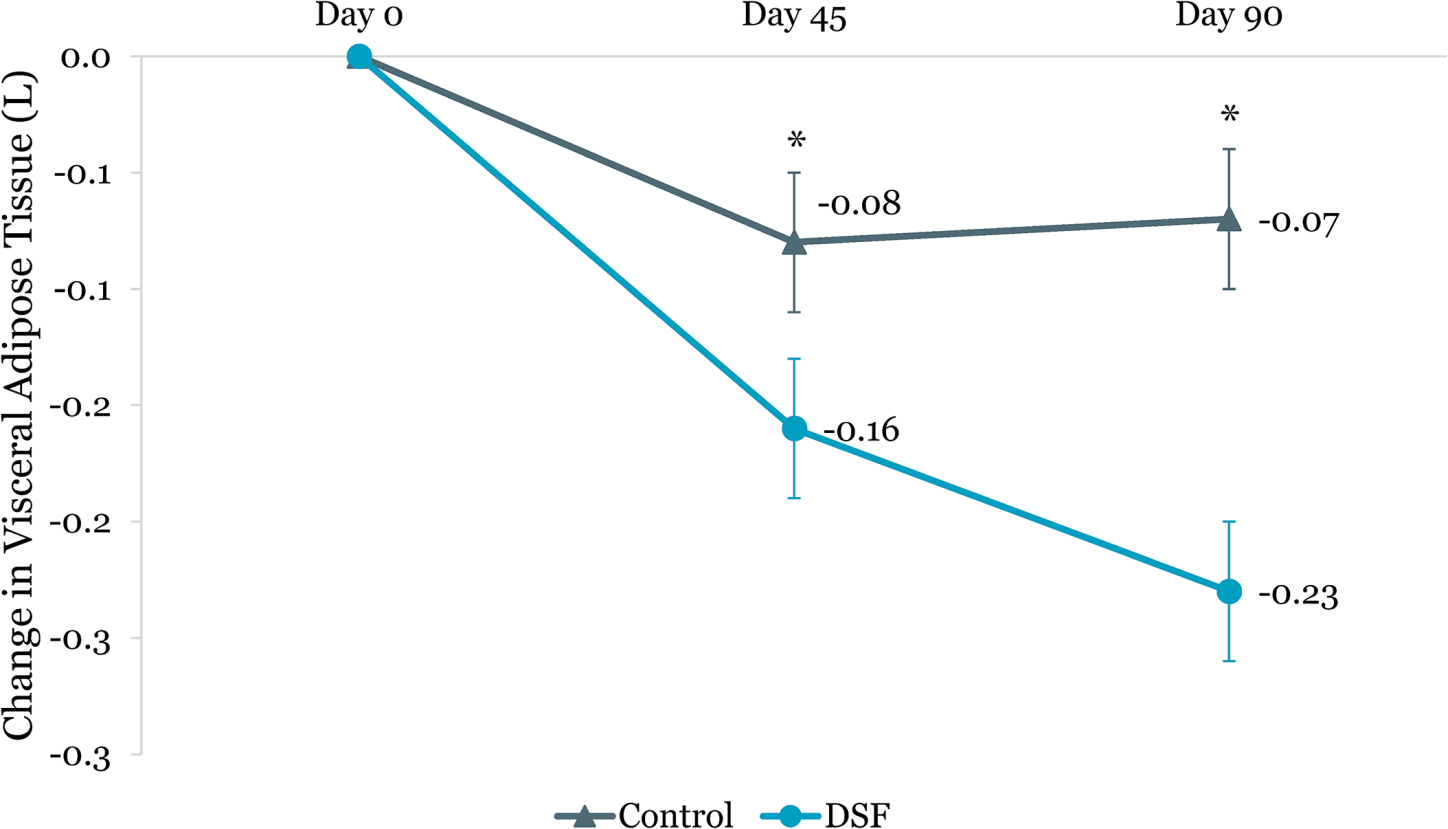
Change in visceral adipose tissue (L) from day 0. Values are LSM ± SE. *Significantly different between the groups (*p <* 0.05). DSF, diabetes-specific formula.

**Figure 11:**
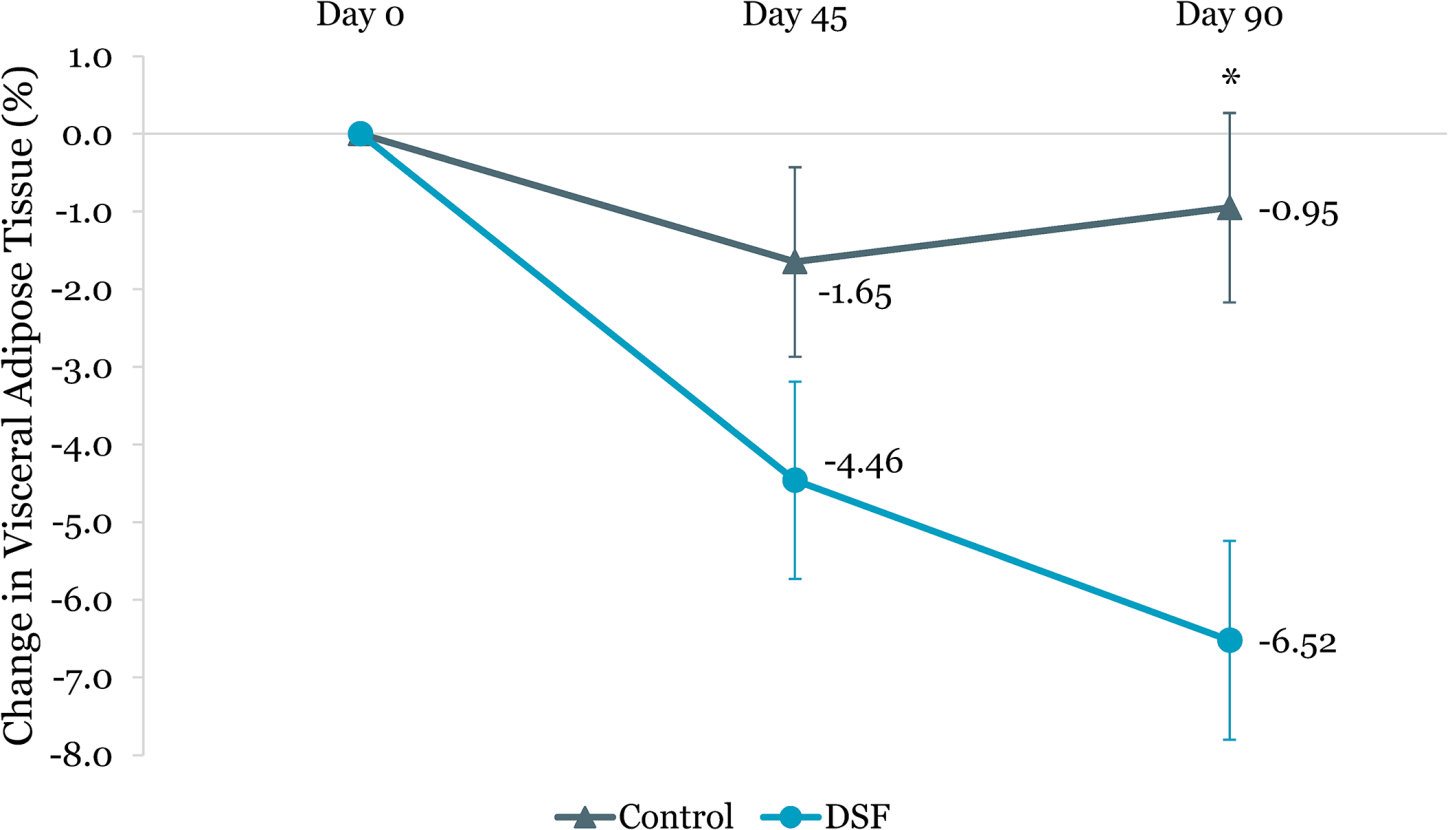
Change in visceral adipose tissue (%) from day 0. Values are LSM ± SE. *Significantly different between the groups (*p <* 0.05). DSF, diabetes-specific formula.

The DSF group was found to have a twofold greater reduction in visceral adipose tissue than the control group at day 45 (−0.16 L vs. −0.08 L, *p =* 0.039) and day 90 (−0.23 L vs. −0.07 L, *p <* 0.001) (Figure 10). Similarly, percent change in visceral adipose tissue in the DSF group was significantly lower than the control group at day 90 (−6.52% vs. –0.95%, *p <* 0.001) (Figure 11).

Further analysis showed a significant difference between the groups in terms of HbA1c change from day 0 to day 90 (overall *p* = 0.006) when the DSF group was split in the analysis as one serving per day or two servings per day. HbA1c level was reduced by 0.21% in the control group, 0.46% in the one serving per day DSF group, and 0.54% in the two servings per day DSF group (Supplementary Figure S1). The difference in HbA1c level changes between the one-DSF and two-DSF groups was not statistically significant (*p* = 0.559). In addition, there was a significant difference in the change in visceral adipose tissue (L) from day 0 to day 90 between the groups (overall *p* = 0.001, Supplementary Figure S2). The reduction in visceral adipose tissue was significantly greater in the one serving per day DSF group (−0.23 L) and the two servings per day DSF group (−0.22 L), compared to the control group (−0.07 L), with no significant difference between the one-DSF and two-DSF groups (*p* = 0.943). Similarly, both DSF groups experienced a significantly greater reduction in the percent change in visceral adipose tissue from day 0 to day 90, compared to the control group (overall *p* = 0.003, Supplementary Figure S3), with no significant difference between the one-DSF and two-DSF groups (*p* = 0.641).

Subgroup analysis in participants with higher baseline BMI (27.5 to <35.0 kg/m^2^) showed significant improvements in triglycerides levels and total cholesterol:HDL cholesterol ratio in the DSF group, compared to the control group. Specifically, DSF group had significantly lower triglycerides levels than the control group at day 45 (−0.09 mmol/L vs. 0.20 mmol/L; *p* = 0.035) and day 90 (−0.06 mmol/L vs. 0.18 mmol/L; *p* = 0.035). Additionally, total cholesterol:HDL cholesterol ratio was significantly lower in the DSF group at day 90 (−0.28 vs. 0.04; *p* = 0.016).

### 3.4 Compliance and hedonic ratings (DSF group)

The degree of compliance was high in both DSF groups, with an overall compliance of 93.8%. Both groups achieved greater than 90% compliance, i.e., 97.9% for the one serving per day group and 90.9% for the two servings per day group (Supplementary Figure S4A). In addition, all study participants (100%) in the one-DSF group and approximately 90% in the two-DSF group consumed ≥75% of the study products over the 90-day period (Supplementary Figure S4B).

The hedonic ratings for the DSF were very high at baseline, mid-study, and end of study. At baseline, over 94% of the study participants in the DSF group stated they liked the study product, i.e., 13.7% “like extremely”, 39.3% “like very much”, 32.5% “like moderately”, and 8.6% “like slightly”. The hedonic ratings remained high at mid-study and end of study. At the end of the study, over 96% of the participants stated that they liked the product, i.e., 11.8% “like extremely”, 50.9% “like very much”, 28.2% “like moderately”, and 5.5% “like slightly”. There was no significant change in hedonic ratings from baseline to end of study (*p* = 0.580). Similarly, no changes in hedonic ratings were seen from baseline to mid-study (*p* = 0.780) and from mid-study to end of study (*p* = 0.758).

### 3.5 Safety evaluation

Treatment-emergent AEs were reported in 21% of the control group and 22% of those in the DSF group. An equal number of AEs (*n* = 33) were reported in both the DSF and control groups, with 66 total AEs being reported. Of the 66 events reported, 65 were non-serious AEs (NSAEs) and one was a serious AE (SAE). The SAE (COVID-19 pneumonia) was reported in the DSF group and was determined to be not related to the interventional product.

Six gastrointestinal intolerance AEs were reported in the DSF group (5.0%), and none were reported in the control group (0.0%). Four preferred terms (PTs) represented the six AEs reported in the DSF group. These included diarrhea (three participants), gastritis (one participant), gastroesophageal reflux disease (GERD) (one participant) and vomiting (one participant). The reported severity of these PTs was mild. The principal investigator deemed a probable relationship to interventional product for two events (one for vomiting and one for diarrhea). All other AEs for gastrointestinal intolerance were determined to be not related to the interventional product. The reported rates for any single PT for gastrointestinal intolerance were neither statistically nor clinically significant.

Overall, no safety concerns were noted, and no statistically significant trends were observed for any PTs in the DSF group.

## 4 Discussion

This study demonstrated that the consumption of DSF as a meal replacement orpartial meal replacement (1–2 servings per day) in addition to SOC over a 90-day period resulted in significantly greater improvements in glycemic control, body composition, anthropometric measurements, and blood pressure than SOC alone in overweight and obese adults with T2D. The DSF group consistently demonstrated greater reductions in HbA1c and body weight than the control group; this difference became apparent by day 45 and persisted until day 90. The magnitude of the reduction was twofold greater in the DSF group than in the control group for both HbA1c (−0.50% vs. –0.21%) and body weight (−2.27% vs. –1.05%) at day 90. In addition, the decrease in percent body fat and increase in fat-free mass at day 90 in the DSF group were almost twice that of the control group (1.44% vs. 0.79%). Compared with the control group, the DSF group also experienced a significantly greater reduction in percent change in visceral adipose tissue at day 90 (−6.52% vs. –0.95%).

To the best of our knowledge, this is the first study to report that a 3-month intervention using DSF in conjunction with SOC significantly improves body composition compared with SOC alone in overweight and obese individuals with T2D in Asia. Specifically, the reduction in body weight observed in the DSF group coincided with a significant increase in percent fat-free mass and significant reductions in fat mass, visceral adipose tissue, and waist and hip circumferences. In this study, the DSF group had substantially lower systolic and diastolic blood pressures than the control group. Elevated HbA1c, obesity, high blood pressure, and dyslipidemia are major risk factors for cardiovascular disease and complications in people with diabetes, and thus addressing these risks can improve clinical outcomes and reduce cardiovascular events and mortality in this population (46).

A reduction of 0.5% in HbA1c is recognized as clinically significant in the management of diabetes (47, 48). This target was achieved in our intervention group within 3 months. Previous research has shown that reducing HbA1c by 0.1% or 0.2% in people with diabetes is associated with a decrease in mortality by 5 and 10%, respectively (49). Furthermore, the United Kingdom Prospective Diabetes Study demonstrated that for every 1% decrease in HbA1c levels, there was a 37% decrease in microvascular complications and a 21% reduction in the risk of any diabetes-related endpoint or death (50). A real-world study designed to assess the efficacy of nine oral glucose-lowering drugs in reducing HbA1c levels in adult Asians with T2D showed that the initiation of oral glucose-lowering drugs led to a reduction in HbA1c, ranging from −0.3 to −1.1%, with more pronounced effects observed with dipeptidyl peptidase 4 (DPP-4) inhibitors (−0.7% to −0.9%) and sodium-glucose cotransporter 2 (SGLT-2) inhibitors (−0.6% to −1.0%) (51). Previous research showed that lifestyle intervention through meal replacement or partial meal replacement may provide similar glycemic control benefits to oral glucose-lowering drugs. In randomized controlled trials (RCTs) and interventional studies assessing various DSFs as meal replacement or partial meal replacement for lifestyle intervention in the T2D population, the change in HbA1c from baseline ranged from −0.50% to −1.10% (30–32, 35, 36). Significantly greater reduction in HbA1c has also been noted with DSFs than with control diets in several studies, whereby the intervention period spanned from 3 months to 6 months (30, 31, 35–37). The changes in fasting blood glucose were variable across these studies, with a few studies demonstrating a significant reduction in fasting blood glucose levels from baseline (31, 32, 36) or a significantly greater reduction in blood glucose levels with DSFs compared with control diets (31, 35, 37). In our study, we observed that the fasting blood glucose was significantly reduced compared with the control group at day 90.

The improvements in HbA1c and fasting blood glucose in our study are consistent with findings in previous studies involving DSFs in individuals with T2D. In a separate RCT by Mottalib et al., overweight and obese adults with T2D undergoing structured nutrition therapy with a DSF (1–3 times daily for 4 months) experienced significant HbA1c reductions of −0.61% and − 0.66%, with and without weekly tele-counselling by a nutritionist, respectively, compared with a 0.06% increase in the control group with individualized nutrition therapy (regular meal planned with the study nutritionist) (30). Another RCT comparing structured Ramadan Nutrition Therapy with a DSF versus SOC in individuals with T2D showed that after 8 weeks of intervention, the DSF group had improved glycemic control (HbA1c and fasting plasma glucose) and enhanced dietary adequacy (42). In a few RCTs where DSFs were used as meal replacement or partial meal replacement as a component of lifestyle intervention (31, 32, 35), HbA1c changes from baseline ranged from −0.5% to −1.1%. Similar trends were noted for changes in fasting blood glucose, both in terms of changes from baseline and between-group differences (31, 32, 35). Notably, the Malaysian tDNA study by Chee et al. evaluated the use of DSF as meal replacement (once or twice daily) compared with usual care (UC); findings at month 6 revealed no significant changes in HbA1c from baseline in the UC group (−0.2%), whereas both tDNA intervention groups exhibited significant improvement (31). The tDNA group receiving motivational interviews showed a greater reduction in HbA1c (−1.1%) than did the tDNA group with conventional counseling (−0.5%) (31). The intervention in our study closely resembles the tDNA group with conventional counseling, and the magnitude of HbA1c changes in our study at day 90 was comparable to that of the Chee et al. study at month 6.

The majority of DSF formulations are characterized as being low calorie, low GI, low carbohydrate, high fiber, high protein, high MUFA and PUFA (22). The nutrient blend and ingredients of DSFs synergistically promote greater satiety and reduce calorie intake, facilitating weight loss. This effect consequently contributes to correcting associated metabolic abnormalities such as hyperglycemia and abnormal cardiometabolic parameters (23). The DSF used in this study comprises a distinct blend of low glycemic carbohydrate system with sucromalt, soluble and insoluble fibers, and high levels of nutrients (such as inositol, vitamin D_3_, zinc, and chromium) to help manage blood glucose levels. It also provides complete and balance nutrition, with essential vitamins and minerals that are often insufficiently supplied in modern diets, for people with diabetes.

The effectiveness of DSF interventions may be influenced by several factors, such as the nature of the intervention (only meal replacement or in combination with physical exercise, health education, or nutrition counseling), the intensity of the intervention (meal replacement or total diet replacement), the duration of the study, and the specific population being studied (individuals with T2D, prediabetes, adults, elderly) (23, 38). The success of any lifestyle intervention, including dietary interventions with DSFs, relies heavily on maximum adherence to the regimen. Although intense lifestyle interventions can lead to favorable results, their feasibility and sustainability over time in real-world situations may be challenging. On the other hand, the seamless integration of a stand-alone DSF as a meal replacement or partial meal replacement into daily routines, may present a more practical approach for individuals seeking sustainable and practical strategies, particularly those who are overweight or obese and with diabetes. Incorporating partial meal replacement can provide individuals with the opportunity to include other nutritious foods in their diet, potentially boosting compliance with their dietary regimen. Furthermore, it has been suggested that, in transitioning from metabolically unhealthy overweight or obesity, adopting a moderate regimen with a focus on gradual weight loss is preferable. This approach may enhance participants’ therapy adherence and minimize dropout rates, because it poses a lower risk of adverse outcomes over time than rapid and significant weight loss (29).

In our study, compliance with both single and double servings of the study DSF was high, at 98 and 91%, respectively, over the 90-day period. This indicates that the intervention can be seamlessly and effortlessly incorporated as a meal replacement or partial meal replacement. Compliance or adherence to DSFs, investigated in limited studies, has been noted to be high, reaching approximately 96% (31, 34). In the Look AHEAD study, participants achieved their maximum weight loss when receiving the most intensive intervention and reporting their highest adherence. Participants in the highest quartile of meal replacement use (608 meal replacements in 1 year) were 4.0 times more likely to reach the 7% weight loss goal and 4.1 times more likely to reach the 10% goal than participants in the lowest quartile (117 meal replacements in 1 year) (52, 53). A study that compared structured Ramadan Nutrition Therapy with a DSF versus SOC in individuals with T2D for 8 weeks showed that with each 1% increase in adherence to the DSF, there was a corresponding reduction of 0.01% in HbA1c levels (42).

Our results indicated no significant difference in insulin levels between the DSF and control groups. These are in line with the findings of previous studies on DSFs as meal replacement in people with T2D, with study periods ranging from 1 month to 3 months (34, 54). A few studies have demonstrated a reduction in insulin levels from baseline after intervention with DSFs (30, 35), and only one study reported statistically significant reduction in insulin levels compared with the control diet (35). The results from RCTs and interventional studies which assessed the effects of DSFs on insulin resistance parameters, such as Homeostatic Model Assessment for Insulin Resistance (HOMA-IR), mostly indicated that DSFs did not lead to improvements in insulin resistance compared with the control group (30, 32, 34). However, variable outcomes may arise due to factors such as differences in DSF formulations, the study population, and the duration and intensity of the study intervention. In the present study, we found that the mean HOMA-β in the DSF group increased from 93.6 at baseline to 109.8 at day 90 whereas the HOMA-β in the control group reduced from 123.9 at baseline to 82.2 at day 90, which represents a significant difference in the change in HOMA-β between the groups (*p* = 0.049). The improvement in HOMA-β in the DSF group by day 90 in this study suggested that DSF may lead to improvement in insulin sensitivity. Long-term studies could provide further insights to validate this potential effect.

In this study, the use of DSF led to significant reductions in body weight and waist and hip circumferences. Achieving a weight reduction of 5–10% in overweight and obese individuals with T2D is considered an optimal therapeutic goal, because it is associated with a reduction of 0.6–1.0% in HbA1c and improvement in other metabolic parameters, with greater benefits observed with increased weight loss (55). Greater weight loss may exert a multifaceted positive impact, improving insulin sensitivity and addressing metabolic dysfunction, ultimately leading to a greater reduction in A1c levels and improvement in various metabolic parameters (56). The majority of previous studies exploring DSFs as meal replacement or partial meal replacement in the T2D population have consistently demonstrated significant reductions in body weight or BMI, relative to baseline (29–33, 36) and control groups (31, 33–36). One interesting finding in our study was that the weight loss was greater in participants who received two servings of DSF per day (−2.97%) versus a single serving per day (−1.44%) or control (−1.10%), suggesting a dose–response relationship between DSF and body weight reduction. Similarly, a previous study demonstrated greater and sustained weight loss for up to 12 months when using two instead of one partial meal replacement a day in adults with T2D (57).

The DSF group in this study showed a significant increase in percent fat-free mass and significant reductions in fat mass, visceral adipose tissue, and waist circumference. These findings suggest that the DSF promotes targeted reduction and positive redistribution of body fat while preserving lean muscle mass. Notably, in this study, there was a significant and twofold greater reduction in visceral adipose tissues in the DSF group than in the control group at both day 45 and day 90. Additionally, the percent change in visceral adipose tissue was significantly lower in the DSF group by day 90 (−6.52% vs. –0.95%). A previous 24-week study conducted in individuals with T2D showed that the visceral fat area improvement rate per 1% body weight reduction was 2.37% with a liquid meal replacement, compared with 1.34% in the conventional diet group (*p* = 0.029) (58). Visceral fat accumulation is associated with insulin resistance and is often reflected in an increased waist circumference and waist-to-hip ratio (59–61). Hence, interventions that are successful at reducing visceral fat may have a pivotal role in reducing the risk of insulin resistance and associated metabolic disorders, which may explain the improvement in HOMA-β observed in this study. Furthermore, weight loss strategies that preserve lean body mass are of great value in individuals who are overweight or obese, to preserve overall physical function (62–64). During weight loss, individuals typically lose both lean and fat mass; however, in this study, DSF offers high-quality protein and essential micronutrients, such as vitamin D, which support muscle health and contribute to the preservation of lean muscle mass.

The positive outcomes related to body weight in our study may be largely attributed to the low caloric content of the DSF (only 228 calories per serving) and to components of the DSF, such as slowly digestible carbohydrates, MUFA and PUFA, high-quality proteins, and fibers. This unique nutrient blend may play a key role in promoting satiety, suppressing appetite, and enhancing fat oxidation (22, 23, 40). Together, these benefits all contribute to the observed improvement in anthropometric measurements and body composition. It is conceivable that a prolonged intervention period beyond 3 months with the DSF in this study may yield the envisaged 5% to 10% body weight reduction, accompanied by corresponding improvements in glycemic control, body composition, and cardiometabolic risk factors.

The levels of triglycerides, total cholesterol, LDL-C, and HDL-C demonstrated no discernible distinctions between the DSF and control groups in the total cohort. These outcomes are consistent with previous investigations evaluating the impact of various DSFs in the T2D population, where lipid levels remained minimally affected (29–32, 34–36, 54). The absence of significant changes in blood lipid levels in this study after the consumption of DSF may be attributed to the fact that many of the study participants were on lipid-lowering medications and their baseline lipid levels were already within the optimal range (65, 66). Interestingly, subgroup analysis showed significant improvements in triglycerides levels at day 45 and day 90, as well as total cholesterol:HDL cholesterol ratio at day 90 in the DSF group, when compared with the control group. Future studies are warranted to understand the potential role of DSF in ameliorating cardiovascular disease risk factors.

In our study, the DSF elicited reductions in systolic and diastolic blood pressures (around 2 mmHg), compared with the control group. A meta-analysis of 25 studies reported a direct correlation between weight loss and blood pressure, i.e., a reduction in weight by 1 kg was associated with approximately 1 mmHg reduction in both systolic and diastolic blood pressures (67). In addition to weight loss, another potential explanation for this outcome is that individuals may reduce their sodium intake by eating out less and replacing meals. Previous studies have shown that dietary sodium intake exceeds the recommended levels in both Malaysia and Thailand (68, 69). Thus, the substitution of conventional meals with DSFs may have clinical significance in the Asian context, particularly regarding blood pressure control.

The DSF used in this study was well tolerated, and thus it can be safely incorporated as a long-term component of medical nutrition therapy for individuals with diabetes who are overweight or obese. Six gastrointestinal-related AEs (5.0%) were observed in the DSF group. The occurrence of mild gastrointestinal symptoms is not uncommon with the use of DSFs (34, 54), and the reported rates for any single PT for gastrointestinal intolerance were neither statistically nor clinically significant.

There are several limitations to consider when interpreting the findings of the present study. First, this study has a relatively short follow-up of 3 months; long-term studies are warranted to confirm the benefits of DSFs as a sustainable meal replacement or partial meal replacement option for overweight and obese individuals with T2D. Secondly, the COVID-19 lockdown restrictions in Malaysia and Thailand led to one potential participant declining to participate in this study after screening, and an additional four out of 235 randomized participants (1.7%) were lost to follow-up during the study. However, sensitivity analysis showed that these missing data have minimal impact on the study findings. Lastly, the pandemic-related restrictions may have influenced participants’ dietary habits and physical activity levels. Thus, confirmation of our findings in non-pandemic times is necessary.

The strengths of this RCT include its assessment of the effect of DSF plus SOC versus SOC only in an adult Asian population with T2D. The study comprehensively addresses diverse outcomes, encompassing glycemic control, body composition, anthropometric measurements, and cardiometabolic health, characterized by robust methodologies and meticulous statistical analyses. In addition, the attrition rate in this study was low, with 229 out of 235 participants completing the study (97.4%). This retention rate is higher than what is reported in most of the previous studies on DSFs in populations with T2D, where the rates of study completion ranged from 73.3 to 95.5% (29, 30, 32, 34, 36, 37). In terms of generalizability, while our study primarily included overweight and obese adults with T2D from the Asian region, the findings may hold relevance beyond this specific demographic.

In line with the findings from previous research (20–23), our study shows that the use of DSF is an important tool to help overweight and obese individuals with T2D to achieve weight loss and to improve glycemic control and cardiometabolic risk factors. The high compliance with the study DSF (94%) suggests that DSF as a meal replacement could potentially be integrated into the lifestyle modification plans with good adherence, to help people with diabetes achieve the goals of nutrition therapy.

## 5 Conclusion

In this study of overweight and obese adults with T2D, the use of DSF as meal replacement or partial meal replacement (1–2 servings per day) in addition to SOC over a 90-day period led to significant reductions in HbA1c level and body weight, with approximately twofold better outcomes than SOC only. To our knowledge, our study is the first to report that a DSF resulted in marked improvement in body composition, characterized by a significant increase in percent fat-free mass and significant reductions in fat mass, visceral adipose tissue, and waist and hip circumferences. The DSF also resulted in significantly lower systolic and diastolic blood pressures than SOC. The study findings are pivotal to enhancing our understanding of the benefits associated with DSFs, thereby paving the way for their potential integration into MNT for individuals with diabetes in Asia.

## Data availability statement

The original contributions presented in the study are included in the article/Supplementary material, further inquiries can be directed to the corresponding author.

## Ethics statement

The studies involving humans were approved by the Malaysia Medical Research and Ethics Committee (NMRR-19-3929-52070) and the Thailand Central Research Ethics Committee (COA-CREC071/2020). The studies were conducted in accordance with the local legislation and institutional requirements. The participants provided their written informed consent to participate in this study.

## Author contributions

SLT: Conceptualization, Data curation, Formal analysis, Methodology, Supervision, Visualization, Writing – original draft, Writing – review & editing. WSSC: Investigation, Writing – review & editing. CD: Investigation, Writing – review & editing. YB: Formal analysis, Visualization, Writing – review & editing. L-LL: Investigation, Writing – review & editing. AB: Investigation, Writing – review & editing. BW: Writing – review & editing. GB: Formal analysis, Methodology, Writing – review & editing. DTTH: Conceptualization, Methodology, Supervision, Writing – review & editing.
