# Supplementary material for: Diabetes-specific formula with standard of care improves glycemic control, body composition, and cardiometabolic risk factors in overweight and obese adults with type 2 diabetes: results from a randomized controlled trial
Source: Front Nutr. 2024 Jul 15;11:1400580. doi: 10.3389/fnut.2024.1400580 (PMC11285196; doi:10.3389/fnut.2024.1400580)
Supplement: Supplementary file 1 [file Data_Sheet_1.docx]

Supplementary Material

Supplementary Table 1. Composition of the study diabetes-specific formula per serving

| Nutrient |  | Unit |  | Diabetes-specific formula |
| --- | --- | --- | --- | --- |
| Energy |  | kcal |  | 228 |
| Protein |  | g |  | 10.2 |
| Fat |  | g |  | 8.7 |
| Carbohydrate |  | g |  | 26.1 |
| Inositol |  | mg |  | 800 |
| Vitamin D_3_ |  | µg |  | 6.51 |
|  |  | IU |  | 261 |
| Vitamin E |  | mg α-TE |  | 5.3 |
|  |  | IU |  | 7.8 |
| Folic acid |  | µg |  | 81 |
| Potassium |  | mg |  | 370 |
| Calcium |  | mg |  | 223 |
| Zinc |  | mg |  | 3.0 |
| Chromium |  | µg |  | 39.6 |
|  |  |  |  |  |

Supplementary Table 2. Baseline body composition, blood pressure, and biochemical outcomes of study participants

| Variables | All participants  (*n* = 235) |  | DSF  (*n* = 117) |  | Control  (*n* = 118) |  | *P* value |
| --- | --- | --- | --- | --- | --- | --- | --- |
| Body composition |  |  |  |  |  |  |  |
| Fat mass (kg) | 27.40 ± 0.45 |  | 27.79 ± 0.64 |  | 27.01 ± 0.64 |  | 0.210 |
| Fat mass (%) | 37.48 ± 0.53 |  | 38.49 ± 0.74 |  | 36.50 ± 0.73 |  | 0.027 |
| Fat-free mass (kg) | 46.03 ± 0.66 |  | 44.55 ± 0.82 |  | 47.46 ± 1.00 |  | 0.020 |
| Fat-free mass (%) | 62.52 ± 0.53 |  | 61.51 ± 0.74 |  | 63.50 ± 0.73 |  | 0.027 |
| Visceral adipose tissue (L) | 3.18 ± 0.08 |  | 3.09 ± 0.11 |  | 3.26 ± 0.12 |  | 0.243 |
| Total body water (%) | 46.28 ± 0.35 |  | 45.59 ± 0.50 |  | 46.95 ± 0.49 |  | 0.026 |
| Phase angle (°) | 5.70 ± 0.00 |  | 5.60 ± 0.10 |  | 5.80 ± 0.10 |  | 0.148 |
| Blood pressure |  |  |  |  |  |  |  |
| Systolic blood pressure (mmHg) | 131.20 ± 0.90 |  | 130.40 ± 1.20 |  | 132.00 ± 1.20 |  | 0.362 |
| Diastolic blood pressure (mmHg) | 79.60 ± 0.60 |  | 79.60 ± 0.90 |  | 79.60 ± 0.80 |  | 0.883 |
| Biochemical outcomes |  |  |  |  |  |  |  |
| Insulin (μIU/mL) | 15.81 ± 0.90 |  | 14.64 ± 0.91 |  | 16.97 ± 1.54 |  | 0.185 |
| HOMA-β | 109.00 ± 17.71 |  | 93.56 ± 10.76 |  | 123.92 ± 33.24 |  | 0.424 |
| HOMA-IR | 5.31 ± 0.31 |  | 4.90 ± 0.35 |  | 5.73 ± 0.50 |  | 0.163 |
| Total cholesterol (mmol/L) | 4.31 ± 0.06 |  | 4.24 ± 0.08 |  | 4.38 ± 0.08 |  | 0.263 |
| Triglycerides (mmol/L) | 1.75 ± 0.07 |  | 1.64 ± 0.09 |  | 1.85 ± 0.10 |  | 0.123 |
| HDL cholesterol (mmol/L) | 1.20 ± 0.02 |  | 1.23 ± 0.03 |  | 1.16 ± 0.02 |  | 0.053 |
| LDL cholesterol (mmol/L) | 2.33 ± 0.05 |  | 2.27 ± 0.07 |  | 2.40 ± 0.08 |  | 0.215 |
| Total cholesterol:HDL cholesterol ratio | 3.78 ± 0.08 |  | 3.65 ± 0.12 |  | 3.92 ± 0.10 |  | 0.088 |

Mean values ± SEM are reported. For some variables, sample sizes are smaller than the overall stated sample sizes. DSF, diabetes-specific formula; HDL, high-density lipoprotein; HOMA-β, homeostasis model assessment of beta-cell function; HOMA-IR, homeostasis model assessment of insulin resistance; LDL, low-density lipoprotein.

**Supplementary Figure 1.** Change in HbA1c (%) from day 0 by treatment group and number of DSF servings

Analysis of covariance (ANCOVA), adjusting for treatment group, site, and baseline value for HbA1c. Stepdown Bonferroni (Holm) *P* value adjustments were made to account for multiple comparisons. DSF, diabetes-specific formula; HbA1c, glycated hemoglobin.

**Supplementary Figure 2.** Change in visceral adipose tissue (L) from day 0 by treatment group and number of DSF servings

Analysis of covariance (ANCOVA), adjusting for treatment group, site, randomization stratum HbA1c level, and baseline value for visceral adipose tissue (L). Stepdown Bonferroni (Holm) *P* value adjustments were made to account for multiple comparisons. DSF, diabetes-specific formula; HbA1c, glycated hemoglobin.

**Supplementary Figure 3.** Change in visceral adipose tissue (%) from day 0 by treatment group and number of DSF servings

Analysis of covariance (ANCOVA), adjusting for treatment group, site, randomization stratum HbA1c level, and baseline value for visceral adipose tissue (%). Stepdown Bonferroni (Holm) *P* value adjustments were made to account for multiple comparisons. DSF, diabetes-specific formula; HbA1c, glycated hemoglobin.

**(A)**

**Supplementary Figure 4.** Study compliance over 90 days in the DSF group (A) and percent of participants by compliance categories (B)

**(B)**

DSF, diabetes-specific formula.
